# Career aspirations of dental students: insights from a multinational study using social cognitive career theory (SCCT)

**DOI:** 10.3389/froh.2025.1577870

**Published:** 2025-04-11

**Authors:** Abanoub Riad, Lamis Elsheikh, Silvi Domnori, Aurora Doris Fratila, Charlotte Carter, Deniz Devrim Kaya, Ekaterina Volevach, Rachael England, Mariana Morgado, Julien Issa, Sameh Attia, Mick Armstrong, Doniphan Hammer, Azamat Baigulakov, IADS Delegates, Aya Abdelrahim, EDSA Delegates, Inès Bouillaud

**Affiliations:** ^1^ International Association of Dental Students (IADS), Geneva, Switzerland; ^2^ Department of Public Health, Faculty of Medicine, Masaryk University, Brno, Czechia; ^3^ Masaryk Centre for Global Health (MCGH), Department of Public Health, Faculty of Medicine, Masaryk University, Brno, Czechia; ^4^ Department of Periodontology, Faculty of Dentistry, Istanbul University, Istanbul, Türkiye; ^5^ Institute of Graduate Studies in Health Sciences, Istanbul University, Istanbul, Türkiye; ^6^ Faculty of Dental Medicine, Ludwig Maximilian University of Munich, Munich, Germany; ^7^ European Dental Students Association (EDSA), Amsterdam, Netherlands; ^8^ International Clinical Research Center (ICRC), St. Anne’s University Hospital, Brno, Czechia; ^9^ World Dental Federation (FDI), Geneva, Switzerland; ^10^ Egas Moniz Center for Interdisciplinary Research (CiiEM), Egas Moniz School of Health & Science, Almada, Portugal; ^11^ Chair of Practical Clinical Dentistry, Department of Diagnostics, Poznań University of Medical Sciences, Poznań, Poland; ^12^ Doctoral School, Poznań University of Medical Sciences, Poznań, Poland; ^13^ Department of Periodontology, Oral Surgery, and Oral Medicine, Charité - Universitätsmedizin Berlin, Berlin, Germany

**Keywords:** career choice, career counselling, continuing dental education, dental societies, dental students, social cognition

## Abstract

**Background:**

Dental students' career choices are shaped by many factors, including their personal abilities and goals, environmental factors and the resources available to them. Understanding the drivers for this career pathway decision is crucial for educational institutions' development of comprehensive curricula. This study applies Social Cognitive Career Theory (SCCT) to investigate the professional aspirations of dental students globally, providing insights into the factors that influence career choices of dental students from different regions to understand how personal, socioeconomic and cultural differences influence their decisions.

**Methods:**

A cross-sectional, multicentred survey was conducted between May and July 2023, involving 1964 dental students from over 20 countries. Self-administered questionnaires based on SCCT were used to assess participants' self-efficacy, professional and personal outcome expectations, career goals, and career path preferences. Statistical analysis, including multivariable logistic regression and mediation analysis, was employed to identify the relationships between the SCCT framework, sociodemographic factors and career aspirations.

**Results:**

The study revealed that 51.2% of participants preferred a specialty in clinical dentistry, while 28.1% aimed for general dentistry. Mediation analysis demonstrated notable pathways from career planning training to career aspirations through self-efficacy, professional and personal expectations. Self-efficacy mediated 26.7%–98.65% of the effect on career preferences coming forward as a key mediator. Demographic statistics demonstrated that regional and economic differences significantly impacted students’ career choice, where students from higher-income countries reported more likely to choose general dentistry and those from lower-income countries were more drawn to specialty fields or public health.

**Conclusions:**

This study offers new insights into the global career aspirations of dental students through the prism of the SCCT. These findings highlight the need for dental schools and associations to offer tailored career planning training based on students' backgrounds at an early phase of their education. Providing support and career guidance, especially in underserved regions, can help students make informed decisions that align with their personal and professional goals. This will ultimately contribute to a more diverse and well-prepared global dental workforce.

## Introduction

Career aspirations encompass the professional objectives individuals establish for themselves, which subsequently inform their decision-making processes and delineate their professional trajectories. Per young adults, career aspirations bear particular significance, as this transitional stage entails the formation of professional identities and the pursuit of designated career paths (1). The examination of young adults' career aspirations yields valuable insights into their motivations, values, and expectations, thereby enabling the development of tailored interventions and support mechanisms to facilitate their professional advancement and achievements (2). Discerning the elements that influence these aspirations additionally facilitates the identification of potential barriers and opportunities for intervention, ensuring that young adults are sufficiently equipped to successfully navigate the intricate and dynamic landscape of professional endeavours (2, 3).

Higher education significantly influences students' career aspirations, including those pursuing dental education (2). While dental schools play a crucial role in equipping students with the requisite knowledge and skills to practice their chosen profession (4, 5), other entities such as dental student organisations, dentist associations and similar NGOs also contribute to shaping their diverse career trajectories, thereby fostering awareness of the extensive opportunities available within the healthcare domain. By collaborating with dental schools, through mentorship programs, career guidance services and professional networks, these organisations provide students with awareness of extensive opportunities within the healthcare domain and support their professional development (5, 6). Altogether this apparatus of institutions guides students in navigating the intricate network of professional endeavours, casting light on the diverse career alternatives and nurturing the competencies necessary for success in these paths (6). Consequently, elucidating the significance of the combined role of dental education institutions, dental professional organizations and student-led NGOs in shaping students' career choices and aspirations is essential for cultivating a well-prepared and motivated workforce, capable of facing the evolving demands of the dental profession and enhancing oral health outcomes for the broader population (7).

The Social Cognitive Career Theory (SCCT) offers an invaluable framework for comprehending dental students' career aspirations. This theory, formulated by Lent, Brown, and Hackett, posits that career development is governed by three principal elements: self-efficacy, outcome expectations, and personal goals (8, 9). Self-efficacy pertains to an individual's conviction in their capacity to successfully execute a particular task or attain a specific outcome. Outcome expectations represent the anticipated repercussions of engaging in a particular behaviour, while personal goals embody an individual's commitment to pursuing a designated career trajectory (10, 11). By employing SCCT to examine dental students' career aspirations, crucial insights can be gained into the factors that motivate and direct their professional selections and pinpoint potential areas for targeted interventions. For instance, augmenting self-efficacy and providing realistic outcome expectations may encourage students to investigate a broader array of career options within the dental field, while fostering the development of well-defined personal goals can support their long-term career success and satisfaction. Moreover, the SCCT framework can assist educators, administrators, and policymakers in dental education institutions to more comprehensively understand the factors driving students' career decisions, empowering them to devise more efficacious and tailored learning environments that foster career development and ultimately enhance the calibre of the dental workforce.

Understanding the career goals of dental students is essential for developing tailored interventions and support systems that foster their success and professional progress (5, 7). Through a variety of direct and indirect channels, dental education institutions have a crucial role in influencing students' career choices and objectives. Career planning training tailored to the specific needs of dental students globally can eventually lead to a more prepared and motivated dental workforce capable of addressing the changing needs of the dental profession and enhancing the general public's oral health outcomes in a broader array (5).

The overarching goal of this study was to examine the career aspirations of dental students from various countries and regions using a self-administered questionnaire grounded in SCCT. The primary objectives were: (a) to explore the relationships between self-efficacy, outcome expectations, and personal goals as outlined in the SCCT framework and their impact on dental students' career aspirations; and (b) to identify key factors influencing these aspirations, including sociodemographic variables and educational experiences. The secondary objectives were: (a) to investigate students' specific career preferences; and (b) to examine their preferred agencies and media for career planning training.

## Materials and methods

### Design

An analytical multicentre cross-sectional survey-based study was conducted between May and June 2023, to explore the career intentions of undergraduate dental students and recent graduates worldwide. The study adhered to the Strengthening the Reporting of Observational Studies in Epidemiology (STROBE) guidelines for cross-sectional studies, and data was collected via an online self-administered questionnaire (SAQ) using KoBoToolbox (Kobo Inc., Cambridge, MA, USA, 2023) (12, 13).

### Setting

This study emerged from a collaborative initiative between the World Dental Federation (FDI), the International Association of Dental Students (IADS), and the European Dental Students Association (EDSA). After protocol finalisation, ethical clearance, and instrument validation, national delegates from IADS and EDSA were briefed about the study. Interested delegates received a single-login URL to access the SAQ, preventing duplicate responses. To enhance representativeness, data collectors used social media, instant messaging, printed materials, and direct emails via IADS and EDSA mailing lists. While efforts were made to engage underrepresented regions through national dental associations and student leaders, the online survey's voluntary nature may have introduced self-selection bias, favouring students more engaged in digital platforms and dental organisations.

### Population

The target population encompassed dental students and those who had graduated recently. Inclusion criteria stipulated that participant should: (a) either be an undergraduate dental student enrolled in a full-time degree programme; be an intern dentist where vocational training was mandatory for professional licensure; or have graduated after 1 January 2020; (b) be at least 18 years of age; and (c) have given their consent to participate in the study. Conversely, the exclusion criteria were: (a) being enrolled in preparatory courses, like pre-dental, or having graduated before 1 January 2020; (b) being younger than 18 years; and (c) not having given consent for study participation.

Given the global scope and the diverse representation from various regions, a pooled analysis approach was utilised. The data from all participating countries were aggregated to ensure a robust analysis. The sample size was determined to achieve a confidence level of 95% with a more precise margin of error of 2.5%, as calculated using Epi Info™ version 7.2.4 (14). The required minimum sample size for this study was 1,532 valid responses.

### Instrument

The SAQ comprised multiple-choice and Likert-like scale items categorised as follows:

(a) sociodemographic characteristics, encompassing gender, age, nationality, country of study, academic year, university type (public vs. private), and study loan (yes vs. no);
(b) self-efficacy regarding the ability to determine one's career path;
(c) outcome expectations (both professional and personal ones) of selecting specific career paths;
(d) goals to be realised through chosen career paths; and
(e) preferred career paths and learning resources to facilitate career planning.

The SAQ was based on the SCCT scale for medical students developed by Rogers et al. to evaluate self-efficacy, outcome expectations and career goals (11). Rogers scale demonstrated robust internal validity. Content validity was achieved by aligning items with the SCCT theoretical model and obtaining feedback from an expert review panel on the relevance of the scale items. Construct validity was further established through confirmatory factor analysis (CFA) ensuring its feasibility for use in subsequent research (11).

To validate the SAQ, a panel of experts in dental practice policy, dental education, health psychology, and dental public health was consulted. These experts provided feedback on the SAQ items. Based on their insights, necessary amendments were made to the items to enhance their relevance and clarity. Moreover, the CFA of SCCT components indicated an acceptable fit of the model to the data with a *χ*^2^/df ratio of 6.28. The Root Mean Square Error of Approximation (RMSEA) was 0.0519, with a 90% confidence interval of 0.0495–0.0542, indicating a reasonable fit. The Comparative Fit Index (CFI) was 0.936, and the Tucker–Lewis Index (TLI) was 0.929, both suggesting acceptable fit. Additionally, the Standardized Root Mean Square Residual (SRMR) was 0.0392, indicating a good fit.

The SAQ was produced primarily in English, but to maximise coverage and reduce sampling bias, versions in Albanian, Arabic, Czech, French, German, Indonesian, Italian, Spanish, and Turkish were also developed. The choice of languages was based on the logistical capabilities of the investigators' team. For each language, two independent forward translations were conducted. A review panel then compared these translations to produce a consensus version for each language (Supplementary Table S1).

### Variables

The socioeconomic categorisation of countries was based on the World Bank (WB) classification: low income, lower middle income, upper middle income, and high income (15). Geographically, countries were classified as per the FDI geopolitical scheme into: Africa, Asia Pacific, Europe, Latin America, and North America (16).

For the outcome variables, self-efficacy was measured using 7 items, each graded on a 5-point Likert-like scale ranging from “Strongly Disagree (1)” to “Strongly Agree (5)”. The overall self-efficacy score was calculated as a composite of all items in this domain, thus ranging between 7 and 35. Similarly, the professional outcome expectations had 8 items with a total score ranging between 8 and 40, and the personal outcome expectations had 4 items with a total score ranging between 4 and 40. Goals had 6 items with a total score ranging between 6 and 30.

### Data quality control

The initial sample comprised 2,293 respondents. Rigorous techniques were employed to remove low-quality entries and ineligible participants (17). The reasons of responses removal included graduation year (*n* = 8), age limitation (*n* = 14), response duration (*n* = 182), and response pattern (*n* = 125). Following these refinements, the final sample consisted of 1,964 respondents (Supplementary Figure S1).

### Ethical considerations

This study was carried out in accordance with the Declaration of Helsinki for research involving human subjects, and it was thoroughly reviewed and approved by the Ethics Committee of the Faculty of Medicine, Masaryk University (decision no. 12/2023). All participants had to provide their informed consent digitally prior to getting access to the SAQ.

### Statistical analyses

Statistical analysis was conducted using SPSS version 28 and the R-based package Jamovi (18, 19). Descriptive statistics used frequencies, percentages, means, and standard deviations. The Shapiro–Wilk test assessed normal distribution with a significance level (*p.*) < 0.05. Inferential statistics, e.g., Chi-squared (*χ^2^*), Mann–Whitney (*U*), Kruskal–Wallis (*H*), multivariable logistic regression, and mediation analyses were performed with a significance level (*p.*) less than 0.05.

## Results

### Sociodemographic characteristics

The study included 1,964 participants, of whom 71.8% were female and 28.2% were male, with a mean age of 22.42 ± 2.85 years. Nearly two-thirds (66.7%) were enrolled in clinical years, and 7% were recent graduates: 0.6% from the class of 2020, 2.4% from 2021, 2.5% from 2022, and 1.5% from 2023. The majority (87%) were domestic students, meaning they were studying in their country of nationality (Table 1).

**Table 1:** Sociodemographic characteristics of dental students participating in FDI–IADS–EDSA survey of career planning, May–July 2023, (*n* = 1,964).

Variable	Outcome	Frequency (%)/Mean ± SD
Gender	Female	1,411 (71.8%)
	Male	553 (28.2%)
Age	Years	22.42 ± 2.85
Academic year	First year	314 (16%)
	Second year	340 (17.3%)
	Third year	361 (18.4%)
	Fourth year	362 (18.4%)
	Fifth year	297 (15.1%)
	Sixth year	101 (5.1%)
	Internship/Vocational Training	51 (2.6%)
	Graduated^╪^	138 (7%)
^╪^Graduation year	2020	11 (0.6%)
	2021	48 (2.4%)
	2022	50 (2.5%)
	2023	29 (1.5%)
Clinical training	Pre-clinical Student	654 (33.3%)
	Clinical Student	1,310 (66.7%)
Nationality	International Student	256 (13%)
	Domestic Student	1,708 (87%)
Economic level	Low Income	74 (3.8%)
	Lower-middle Income	308 (15.7%)
	Upper-middle Income	627 (31.9%)
	High Income	955 (48.6%)
Region	Africa	284 (14.5%)
	Asia Pacific	174 (8.9%)
	Europe	1,357 (69.1%)
	South America	128 (6.5%)
	North America	21 (1.1%)
Financial aid	No Aid	1,520 (77.4%)
	Aid Received	444 (22.6%)
Career planning training	Not Received	1,389 (70.7%)
	Received	575 (29.3%)

According to the World Bank classification, 48.6% studied in high-income countries, 31.9% in upper-middle-income countries, 15.7% in lower-middle-income countries, and 3.8% in low-income countries. Most participants were studying in Europe (69.1%), followed by Africa (14.5%) and the Asia Pacific region (8.9%). Financial aid was received by 22.6% of the participants, and 29.3% reported receiving career planning training (Supplementary Table S2).

### Self-efficacy in career planning

When asked about their confidence in choosing a career path, 25.6% of participants expressed uncertainty about selecting a career that would fulfil their expectations and goals, 24.8% were unsure about choosing a career that would fit their personality type, 25.2% doubted their ability to choose a career that would enable them to live the desired lifestyle, and 21.4% were uncertain about selecting a career that aligned with their interests and abilities. Furthermore, 44.9% were unsure about what they were willing to sacrifice for their career path, 24.6% could not determine what they valued most in a medical career, and 42.6% struggled to find valid and accurate information for making their career choice. The mean score of the self-efficacy subscale was 26.75 ± 4.67 (range: 7–35) (Table 2).

**Table 2:** Self-efficacy, professional expectations, personal expectations and career goals of dental students participating in FDI–IADS–EDSA survey of career planning, May–July 2023, (*n* = 1,964).

Item		Strongly agree (=5)	Rather agree (=4)	Not sure (=3)	Rather disagree (=2)	Strongly disagree (=1)	Total (µ ± SD)
Self-efficacy	How confident are you at this stage of your training that you could…?						
	Choose a career path that will fulfil your expectations and goals.	469 (23.9%)	992 (50.5%)	355 (18.1%)	122 (6.2%)	26 (1.3%)	3.89 ± 0.88
	Choose a career path that will fit well with your personality (e.g., being an extrovert/introvert).	496 (25.3%)	981 (49.9%)	338 (17.2%)	120 (6.1%)	29 (1.5%)	3.91 ± 0.89
	Choose a career path that will enable you to live the type of lifestyle you desire.	605 (30.8%)	865 (44.0%)	318 (16.2%)	134 (6.8%)	42 (2.1%)	3.95 ± 0.97
	Choose a career path that will fit your interests and abilities.	577 (29.4%)	966 (49.2%)	275 (14.0%)	119 (6.1%)	27 (1.4%)	3.99 ± 0.90
	Decide what you are and are not ready to sacrifice in order to choose a career path.	334 (17.0%)	749 (38.1%)	578 (29.4%)	258 (13.1%)	45 (2.3%)	3.54 ± 0.99
	Decide what you value most in a medical career (e.g., relationships with patients, prestige, or technical skills, etc.).	506 (25.8%)	974 (49.6%)	333 (17.0%)	116 (5.9%)	35 (1.8%)	3.92 ± 0.90
	Locate valid and accurate information to help you choose between equally desirable specialties.	313 (15.9%)	815 (41.5%)	528 (26.9%)	253 (12.9%)	55 (2.8%)	3.55 ± 1.00
	Overall Score (Range: 7–35)						26.75 ± 4.67
Professional expectations	When thinking about the type of career path you are interested in (e.g., clinical practice, academia, public health), how much do you expect at this stage of your training that your choice of career path will…?						
	Be intellectually stimulating.	578 (29.4%)	1,036 (52.7%)	241 (12.3%)	83 (4.2%)	26 (1.3%)	4.05 ± 0.84
	Provide you with work satisfaction.	668 (34.0%)	975 (49.6%)	222 (11.3%)	78 (4.0%)	21 (1.1%)	4.12 ± 0.83
	Allow you to interact with your colleagues.	563 (28.7%)	985 (50.2%)	303 (15.4%)	88 (4.5%)	25 (1.3%)	4.00 ± 0.86
	Let you practice clinical skills that best suit your perceived abilities.	684 (34.8%)	928 (47.3%)	236 (12.0%)	90 (4.6%)	26 (1.3%)	4.10 ± 0.87
	Provide you with a good income.	721 (36.7%)	809 (41.2%)	290 (14.8%)	102 (5.2%)	42 (2.1%)	4.05 ± 0.96
	Allow you to perform a broad spectrum of work.	484 (24.6%)	918 (46.7%)	420 (21.4%)	122 (6.2%)	20 (1.0%)	3.88 ± 0.89
	Be compatible with your interests.	596 (30.3%)	967 (49.2%)	289 (14.7%)	80 (4.1%)	32 (1.6%)	4.03 ± 0.87
	Allow you to achieve your desired professional success.	711 (36.2%)	850 (43.3%)	293 (14.9%)	81 (4.1%)	29 (1.5%)	4.09 ± 0.90
	Overall Score (Range: 8–40)						32.31 ± 5.19
Personal expectations	When thinking about the type of career path you are interested in (e.g., clinical practice, academia, public health), how much do you expect at this stage of your training that your choice of career path will…?						
	Allow you to work the number of hours that you desire.	432 (22.0%)	748 (38.1%)	453 (23.1%)	270 (13.7%)	61 (3.1%)	3.62 ± 1.07
	Allow you to pursue leisure time activities/interests that you like.	399 (20.3%)	807 (41.1%)	453 (23.1%)	258 (13.1%)	47 (2.4%)	3.64 ± 1.02
	Allow you to have your desired work/recreational balance.	404 (20.6%)	797 (40.6%)	481 (24.5%)	246 (12.5%)	36 (1.8%)	3.66 ± 1.00
	Allow you to have your desired lifestyle.	518 (26.4%)	842 (42.9%)	410 (20.9%)	152 (7.7%)	42 (2.1%)	3.84 ± 0.97
	Overall Score (Range: 4–20)						14.75 ± 3.56
Career goals	When you think about the type of career path that you might choose (e.g., clinical practice, academia, public health), please indicate if, at this stage of your training, you agree or disagree with the following statements:						
	I have a clear set of goals for my future with regard to choosing a career path.	472 (24.0%)	755 (38.4%)	445 (22.7%)	219 (11.2%)	73 (3.7%)	3.68 ± 1.07
	I have discussed my goals in relation to my career path choice with my family/partner.	556 (28.3%)	766 (39.0%)	318 (16.2%)	256 (13.0%)	68 (3.5%)	3.76 ± 1.10
	I am taking the steps needed to achieve my goal of choosing a career path.	902 (45.9%)	137 (7.0%)	366 (18.6%)	525 (26.7%)	34 (1.7%)	3.69 ± 1.33
	I have examined my interests, values, and abilities in detail to come up with my goal of choosing a career path.	425 (21.6%)	842 (42.9%)	452 (23.0%)	203 (10.3%)	42 (2.1%)	3.72 ± 0.99
	I have a set time frame in which to make a decision about my choice of career path.	339 (17.3%)	666 (33.9%)	455 (23.2%)	348 (17.7%)	156 (7.9%)	3.35 ± 1.19
	I am getting lots of support to achieve my goal of choosing a career path.	572 (29.1%)	689 (35.1%)	411 (20.9%)	204 (10.4%)	88 (4.5%)	3.74 ± 1.12
	Overall Score (Range: 6–30)						21.93 ± 3.91

No statistically significant difference was found in self-efficacy scores between female (26.67 ± 4.74) and male (26.97 ± 4.49) participants, or between students aged ≤ 22 years (26.65 ± 4.78) and those >22 years (26.90 ± 4.51). However, international students (27.95 ± 4.69), pre-clinical students (26.99 ± 4.87), and those who received career planning training (28.47 ± 4.50) had significantly higher self-efficacy scores (*p.* < 0.001, *p.* = 0.046, and *p.* < 0.001, respectively) than domestic students (26.58 ± 4.64), clinical students (26.64 ± 4.57), and those who did not receive career planning training (26.04 ± 4.55) (Table 3).

**Table 3:** Overall scores of self-efficacy, professional expectations, personal expectations and career goals among dental students participating in FDI–IADS–EDSA survey of career planning, stratified by sociodemographic characteristics, May–July 2023, (*n* = 1,964).

Variable	Outcome	Self-efficacy	*p.*	Professional expectations	*p.*	Personal expectations	*p.*	Career goals	*p.*
Gender	Female	26.67 ± 4.74	0.386	32.35 ± 5.20	0.458	14.71 ± 3.58	0.524	21.90 ± 3.92	0.732
	Male	26.97 ± 4.49		32.18 ± 5.16		14.86 ± 3.52		21.98 ± 3.88	
Age group	≤22 years	26.65 ± 4.78	0.327	32.39 ± 5.10	0.472	14.85 ± 3.51	0.179	21.86 ± 3.96	0.411
	>22 years	26.90 ± 4.51		32.18 ± 5.30		14.61 ± 3.63		22.01 ± 3.83	
Academic year	First year	27.21 ± 5.03	0.123	33.15 ± 4.57	**0.039**	15.54 ± 3.08	**<0.001**	22.61 ± 3.76	**0.006**
	Second year	26.79 ± 4.71		32.43 ± 4.98		15.02 ± 3.42		22.10 ± 3.74	
	Third year	26.35 ± 5.04		32.18 ± 5.58		14.63 ± 3.68		21.47 ± 4.05	
	Fourth year	26.90 ± 4.22		32.01 ± 5.04		14.42 ± 3.64		21.85 ± 3.85	
	Fifth year	26.56 ± 4.53		31.78 ± 5.35		14.33 ± 3.72		21.53 ± 4.07	
	Sixth year	27.27 ± 4.49		33.02 ± 5.59		15.16 ± 3.38		21.97 ± 3.61	
	Internship	26.02 ± 4.96		31.80 ± 5.09		14.55 ± 3.38		21.59 ± 4.52	
	Graduated^╪^	26.64 ± 4.06		31.99 ± 5.53		14.14 ± 3.95		22.30 ± 3.85	
^╪^Graduation year	2020	28.73 ± 2.94	0.268	33.82 ± 5.06	0.306	14.45 ± 4.87	0.216	24.82 ± 2.44	0.078
	2021	25.92 ± 4.43		30.75 ± 5.93		13.27 ± 3.76		22.19 ± 4.05	
	2022	26.62 ± 3.99		32.58 ± 5.69		14.40 ± 4.19		21.52 ± 4.04	
	2023	27.07 ± 3.73		32.34 ± 4.47		15.00 ± 3.34		22.86 ± 3.19	
Clinical training	Pre-clinical Student	26.99 ± 4.87	**0.046**	32.77 ± 4.80	**0.018**	15.27 ± 3.27	**<0.001**	22.34 ± 3.76	**0.001**
	Clinical Student	26.64 ± 4.57		32.07 ± 5.36		14.49 ± 3.68		21.72 ± 3.97	
Nationality	International Student	27.95 ± 4.69	**<0.001**	32.89 ± 5.19	**0.013**	15.46 ± 3.66	**<0.001**	22.65 ± 3.75	**<0.001**
	Domestic Student	26.58 ± 4.64		32.22 ± 5.18		14.64 ± 3.54		21.82 ± 3.92	
Economic level	Low Income	26.47 ± 5.11	**<0.001**	31.68 ± 5.64	**<0.001**	15.00 ± 3.53	**0.006**	20.57 ± 4.94	**<0.001**
	Lower-middle Income	25.90 ± 4.77		30.83 ± 5.78		14.02 ± 3.82		20.88 ± 4.20	
	Upper-middle Income	27.52 ± 4.56		32.79 ± 5.38		14.83 ± 3.53		22.56 ± 3.70	
	High Income	26.55 ± 4.60		32.51 ± 4.72		14.91 ± 3.48		21.95 ± 3.76	
Region	Africa	26.09 ± 4.87	**<0.001**	31.32 ± 5.79	**<0.001**	14.23 ± 3.84	**<0.001**	20.68 ± 4.41	**<0.001**
	Asia Pacific	26.04 ± 4.84		31.09 ± 5.65		14.64 ± 3.49		21.39 ± 4.06	
	Europe	26.87 ± 4.60		32.46 ± 4.93		14.68 ± 3.51		22.12 ± 3.73	
	South America	28.10 ± 4.34		34.29 ± 4.67		16.69 ± 2.96		23.16 ± 3.40	
	North America	26.05 ± 5.23		33.38 ± 7.05		15.48 ± 3.28		22.86 ± 5.12	
Financial aid	No Aid	26.66 ± 4.63	0.052	32.18 ± 5.26	0.093	14.68 ± 3.59	0.110	21.84 ± 3.90	**0.026**
	Aid Received	27.07 ± 4.80		32.73 ± 4.90		15.01 ± 3.46		22.22 ± 3.94	
Career Planning Training	Not Received	26.04 ± 4.55	**<0.001**	31.77 ± 5.11	**<0.001**	14.46 ± 3.56	**<0.001**	21.28 ± 3.92	**<0.001**
	Received	28.47 ± 4.50		33.61 ± 5.14		15.46 ± 3.49		23.48 ± 3.42	

Mann–Whitney (*U*) and Kruskal–Wallis (*H*) tests were used with a significance level (*p*.) < 0.05.

Bold font refers to the values that are statistically significant.

### Professional expectations

Regarding their professional expectations, 82.2% of participants believed their chosen career path would be intellectually stimulating, while 83.7% anticipated it would offer work satisfaction. Additionally, 78.8% expected their career to facilitate interaction with colleagues, and 82.1% felt it would allow them to practice clinical skills aligned with their perceived abilities. Furthermore, 77.9% expected their career to provide a good income, 71.4% believed it would enable them to perform a wide range of work, 79.6% felt it would be compatible with their interests, and 79.5% anticipated it would help them achieve their desired professional success. The mean score of the professional expectations subscale was 32.31 ± 5.19 (range: 8–40) (Table 2).

There was no statistically significant difference in professional expectations scores across gender (*p.* = 0.458) or age groups (*p*. = 0.472). However, international students (32.89 ± 5.19), pre-clinical students (32.77 ± 4.80), and those who received career planning training (33.61 ± 5.14) demonstrated significantly higher professional expectations scores (*p.* = 0.013, *p.* = 0.018, and *p.* < 0.001, respectively) compared to domestic students (32.22 ± 5.18), clinical students (32.07 ± 5.36), and those without career planning training (31.77 ± 5.11) (Table 3).

### Personal expectations

In terms of personal expectations, 60.1% of participants believed their chosen career path would allow them to work their desired number of hours. Additionally, 61.4% expected it would enable them to engage in leisure activities and interests they enjoy, 61.2% anticipated achieving a satisfactory work-life balance, and 69.2% felt it would support their desired lifestyle. The mean score of the personal expectations subscale was 14.75 ± 3.56 (range: 4–20) (Table 2).

There was no statistically significant difference in personal expectations scores between genders (*p.* = 0.524) or age groups (*p.* = 0.179). However, international students (15.46 ± 3.66), pre-clinical students (15.27 ± 3.27), and those who received career planning training (15.46 ± 3.49) had significantly higher scores (all *p.* < 0.001) compared to domestic students (14.64 ± 3.54), clinical students (14.49 ± 3.68), and those without career planning training (14.46 ± 3.56) (Table 3).

### Career goals

In evaluating their career goals, 62.5% of participants reported having a clear set of goals for their future career path, and 67.3% had discussed these goals with their families or partners. Moreover, 52.9% had taken the necessary steps to achieve their career goals. Additionally, 64.5% had examined their interests, values, and abilities to determine their career path, 51.2% had established a timeframe for making their decision, and 64.2% were receiving substantial support in pursuing their goals. The mean score of the career goals subscale was 21.93 ± 3.91 (range: 6–30) (Table 2).

There was no statistically significant difference in career goals scores between genders (*p* = 0.732) or age groups (*p* = 0.411). However, international students (22.65 ± 3.75), pre-clinical students (22.34 ± 3.76), and those who received career planning training (23.48 ± 3.42) and financial aid (22.22 ± 3.94) had significantly higher scores (*p.* < 0.001, *p.* = 0.001, *p.* < 0.001, and *p.* = 0.026, respectively) compared to domestic students (21.82 ± 3.92), clinical students (21.72 ± 3.97), and those without career planning training (21.28 ± 3.92) or financial aid (21.84 ± 3.90) (Table 3).

### Career preferences

The most preferred career path among participants was specialty clinical dentistry (51.2%), followed by general clinical dentistry (28.1%), business/entrepreneurship (4.2%), academia (3.6%), and public health (2.2%), with approximately 10.7% undecided. If their first preference was not achievable, the second most preferred paths were specialty clinical dentistry (28.6%), general clinical dentistry (24%), academia (13.5%), business/entrepreneurship (12.7%), and public health (6.6%), while around 14.7% did not have a secondary preference (Table 4).

**Table 4:** Career preferences, and career planning training agencies and preferred Media as reported by dental students participating in FDI–IADS–EDSA survey of career planning, May–July 2023, (*n* = 1,964).

Variable	Outcome	Frequency (%)
Which career path is your first preference upon graduation?	Clinical Dentistry (General Practice)	551 (28.1%)
	Clinical Dentistry (Specialty Practice)	1,006 (51.2%)
	Business/Entrepreneurship	83 (4.2%)
	Academia	70 (3.6%)
	Public Health (e.g., Gov, NGOs, IGOs)	44 (2.2%)
	Undecided	210 (10.7%)
Which career path is your second preference upon graduation?	Clinical Dentistry (General Practice)	421 (24%)
	Clinical Dentistry (Specialty Practice)	502 (28.6%)
	Business/Entrepreneurship	223 (12.7%)
	Academia	236 (13.5%)
	Public Health (e.g., Gov, NGOs, IGOs)	115 (6.6%)
	Undecided	257 (14.7%)
In your opinion, which organization(s) should be responsible for providing career guidance and information to dental students as they plan their professional futures?	Dental Schools	1,765 (89.9%)
	World Dental Federation (FDI)	647 (32.9%)
	Regional Dental Associations	472 (24%)
	National Dental Associations	781 (39.8%)
	International Association of Dental Students (IADS)	639 (32.5%)
	Regional Dental Students Associations (e.g., EDSA)	515 (26.2%)
	National Dental Students Associations	762 (38.8%)
What types of information source(s) do you prefer to use for planning your dental career?	Virtual Sources: Webinars	988 (50.3%)
	Virtual Sources: Podcasts	590 (30%)
	Virtual Sources: Blogs	481 (24.5%)
	Virtual Sources: E-books	466 (23.7%)
	In-person Sources: Continuous Education Workshops	1,207 (61.5%)
	In-person Sources: Undergraduate Elective Courses	1,236 (62.9%)
At what point in their undergraduate education do you think dental students should be introduced to information about various career paths in dentistry?	Upon entry (first years of dental school)	972 (49.5%)
	At the beginning of clinical training (e.g., 3rd/4th yr.)	314 (16%)
	Towards end (senior years of dental school)	678 (34.5%)
Do you think continuous education programs (CE) should have components about career management?	Yes	189 (84.7%)
	Unsure	23 (12.2%)
	No	6 (3.2%)

General clinical dentistry was significantly more common among participants aged over 22 years (32.5% vs. 24.8%; *p.* < 0.001) and clinical students (29.5% vs. 25.2%; *p.* = 0.049) compared to their counterparts. Students from high-income countries (34.3%) and North America (42.9%) were the most likely to prefer general clinical dentistry. Conversely, students from low-income countries (60.8%) and South America (67.2%) were more inclined to prefer specialty clinical dentistry. No statistically significant differences were observed in the preference for general or specialty clinical dentistry based on gender, nationality, financial aid, or prior career planning training (Table 5).

**Table 5:** First career preferences of dental students participating in FDI–IADS–EDSA survey of career planning, stratified by sociodemographic characteristics, May – July 2023, (*n* = 1,964).

Variable	Outcome	General dentistry	*p.*	Specialty dentistry	*p.*	Business	*p.*	Academia	*p.*	Public Health	*p.*
Gender	Female	395 (28.0%)	0.924	732 (51.9%)	0.353	44 (3.1%)	**<0.001**	50 (3.5%)	0.937	28 (2.0%)	0.221
	Male	156 (28.2%)		274 (49.5%)		39 (7.1%)		20 (3.6%)		16 (2.9%)	
Age group	≤22 years	284 (24.8%)	**<0.001**	593 (51.9%)	0.491	51 (4.5%)	0.540	44 (3.8%)	0.421	24 (2.1%)	0.619
	>22 years	267 (32.5%)		413 (50.3%)		32 (3.9%)		26 (3.2%)		20 (2.4%)	
Academic year	First year	78 (24.8%)	**<0.001**	173 (55.1%)	**0.003**	9 (2.9%)	0.185	7 (2.2%)	0.119	2 (0.6%)	0.506
	Second year	87 (25.6%)		160 (47.1%)		23 (6.8%)		14 (4.1%)		9 (2.6%)	
	Third year	86 (23.8%)		192 (53.2%)		18 (5.0%)		16 (4.4%)		9 (2.5%)	
	Fourth year	114 (31.5%)		184 (50.8%)		14 (3.9%)		12 (3.3%)		9 (2.5%)	
	Fifth year	112 (37.7%)		129 (43.4%)		8 (2.7%)		7 (2.4%)		9 (3.0%)	
	Sixth year	35 (34.7%)		50 (49.5%)		3 (3.0%)		2 (2.0%)		2 (2.0%)	
	Internship	7 (13.7%)		33 (64.7%)		3 (5.9%)		5 (9.8%)		1 (2.0%)	
	Graduated^╪^	32 (23.2%)		85 (61.6%)		5 (3.6%)		7 (5.1%)		3 (2.2%)	
^╪^Graduation year	2020	2 (18.2%)	0.788	7 (63.6%)	0.752	1 (9.1%)	0.367	0 (0.0%)	0.534	0 (0.0%)	1.000
	2021	10 (20.8%)		32 (66.7%)		1 (2.1%)		3 (6.3%)		1 (2.1%)	
	2022	14 (28.0%)		28 (56.0%)		1 (2.0%)		4 (8.0%)		1 (2.0%)	
	2023	6 (20.7%)		18 (62.1%)		2 (6.9%)		0 (0.0%)		1 (3.4%)	
Clinical training	Pre-clinical Student	165 (25.2%)	**0.049**	333 (50.9%)	0.849	32 (4.9%)	0.299	21 (3.2%)	0.551	11 (1.7%)	0.237
	Clinical Student	386 (29.5%)		673 (51.4%)		51 (3.9%)		49 (3.7%)		33 (2.5%)	
Nationality	International Student	82 (32.0%)	0.129	135 (52.7%)	0.604	11 (4.3%)	0.952	3 (1.2%)	**0.027**	2 (0.8%)	0.091
	Domestic Student	469 (27.5%)		871 (51.0%)		72 (4.2%)		67 (3.9%)		42 (2.5%)	
Economic level	Low Income	11 (14.9%)	**<0.001**	45 (60.8%)	**<0.001**	2 (2.7%)	0.919	1 (1.4%)	**<0.001**	9 (12.2%)	**<0.001**
	Lower-middle Income	68 (22.1%)		147 (47.7%)		13 (4.2%)		29 (9.4%)		12 (3.9%)	
	Upper-middle Income	144 (23.0%)		358 (57.1%)		29 (4.6%)		22 (3.5%)		14 (2.2%)	
	High Income	328 (34.3%)		456 (47.7%)		39 (4.1%)		18 (1.9%)		9 (0.9%)	
Region	Africa	60 (21.1%)	**<0.001**	133 (46.8%)	**<0.001**	13 (4.6%)	0.682	23 (8.1%)	**<0.001**	17 (6.0%)	**<0.001**
	Asia Pacific	31 (17.8%)		100 (57.5%)		5 (2.9%)		9 (5.2%)		7 (4.0%)	
	Europe	431 (31.8%)		678 (50.0%)		61 (4.5%)		34 (2.5%)		13 (1.0%)	
	South America	20 (15.6%)		86 (67.2%)		3 (2.3%)		3 (2.3%)		6 (4.7%)	
	North America	9 (42.9%)		9 (42.9%)		1 (4.8%)		1 (4.8%)		1 (4.8%)	
Financial aid	No Aid	420 (27.6%)	0.440	783 (51.5%)	0.633	63 (4.1%)	0.740	47 (3.1%)	**0.037**	34 (2.2%)	0.985
	Aid Received	131 (29.5%)		223 (50.2%)		20 (4.5%)		23 (5.2%)		10 (2.3%)	
Career planning training	Not Received	380 (27.4%)	0.285	701 (50.5%)	0.299	60 (4.3%)	0.749	52 (3.7%)	0.505	36 (2.6%)	0.102
	Received	171 (29.7%)		305 (53.0%)		23 (4.0%)		18 (3.1%)		8 (1.4%)	

Chi-squared (*χ^2^*) and Fisher's exact tests were used with a significance level (*p*.) < 0.05.

Bold font refers to the values that are statistically significant.

**Figure 1:**
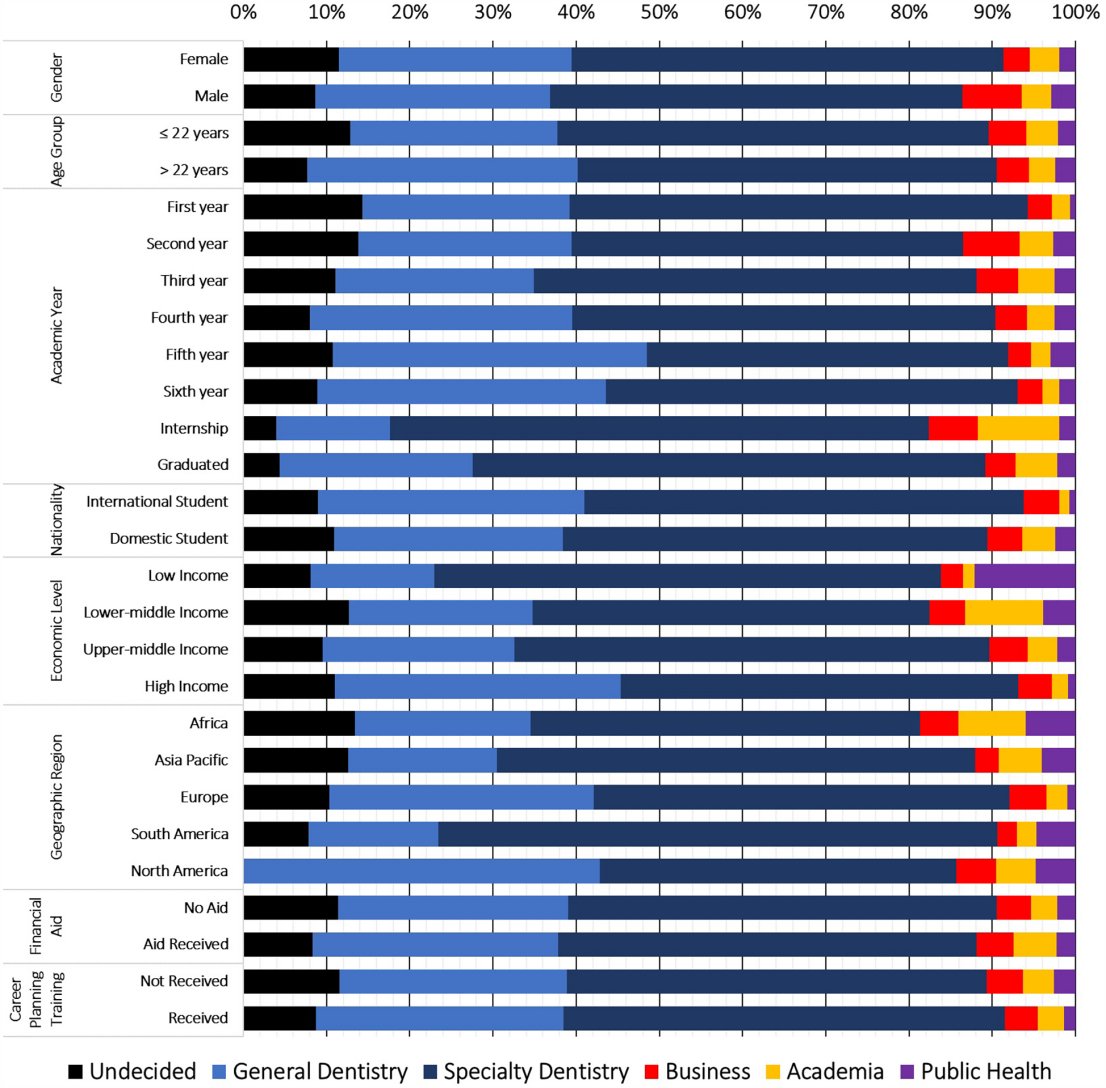
Primary career preferences among dental students participating in the FDI–IADS–EDSA survey of career planning, May–July 2023, (*n* = 1,964).

Business/entrepreneurship was significantly more popular among male students (7.1%) compared to female students (3.1%; *p.* < 0.001). Academia was more commonly preferred by domestic students (3.9% vs. 1.2%; *p.* = 0.027) and those receiving financial aid (5.2% vs. 3.1%; *p.* = 0.037). Students from lower-middle-income countries (9.4%) and Africa (8.1%) were the most likely to choose academia as their primary career path. Regarding the public health career path, students from low-income countries (12.2%) were the most likely to prefer it, followed by those from lower-middle-income (3.9%), upper-middle-income (2.2%), and high-income countries (0.9%). Africa was the region most likely to favour the public health path (6%), while Europe was the least likely (1%; *p.* < 0.001). (Figure 1).

### Career planning training agencies and preferred media

Dental schools (89.9%) were the most frequently cited agency responsible for providing career planning training, followed by national dental associations (39.8%), national dental students associations (38.8%), the FDI (32.9%), the IADS (32.5%), regional dental students associations (26.2%), and regional dental associations (24%) (Table 4).

Dental schools were more commonly preferred by students from high-income countries (95%), North America (95.2%), and those who had not previously received career planning training (91.1% vs. 87%, *p.* = 0.006). Conversely, the FDI was more commonly preferred by those who had received prior career planning training (38.1% vs. 30.8%, *p.* = 0.002). The IADS was more commonly preferred by participants from low-income countries (41.9%) and South America (47.7%), as well as those receiving financial aid (36.7% vs. 31.3%, *p.* = 0.033) (Table 6).

**Table 6:** Career planning training agencies and preferred Media as reported by dental students participating in FDI–IADS–EDSA survey of career planning, stratified by sociodemographic characteristics, May – July 2023, (*n* = 1,964).

Variable	Outcome	Agencies of career planning training									
		Dental Schools	*p.*	FDI	*p.*	Reg. dental associations	*p.*	Nat. dental associations	*p.*	IADS	*p.*
Gender	Female	1,276 (90.4%)	0.185	481 (34.1%)	0.084	350 (24.8%)	0.201	545 (38.6%)	0.099	490 (34.7%)	**<0.001**
	Male	489 (88.4%)		166 (30%)		122 (22.1%)		236 (42.7%)		149 (26.9%)	
Age group	≤22 years	1,024 (89.6%)	0.629	388 (33.9%)	0.265	273 (23.9%)	0.856	455 (39.8%)	0.964	385 (33.7%)	0.200
	>22 years	741 (90.3%)		259 (31.5%)		199 (24.2%)		326 (39.7%)		254 (30.9%)	
Academic year	First year	295 (93.9%)	**<0.001**	107 (34.1%)	0.125	78 (24.8%)	0.386	135 (43.0%)	0.500	99 (31.5%)	0.499
	Second year	296 (87.1%)		111 (32.6%)		70 (20.6%)		124 (36.5%)		97 (28.5%)	
	Third year	312 (86.4%)		131 (36.3%)		102 (28.3%)		150 (41.6%)		131 (36.3%)	
	Fourth year	323 (89.2%)		105 (29.0%)		86 (23.8%)		139 (38.4%)		113 (31.2%)	
	Fifth year	282 (94.9%)		95 (32.0%)		66 (22.2%)		124 (41.8%)		103 (34.7%)	
	Sixth year	94 (93.1%)		30 (29.7%)		22 (21.8%)		43 (42.6%)		35 (34.7%)	
	Internship	47 (92.2%)		25 (49.0%)		11 (21.6%)		18 (35.3%)		18 (35.3%)	
	Graduated^╪^	116 (84.1%)		43 (31.2%)		37 (26.8%)		48 (34.8%)		43 (31.2%)	
^╪^Graduation year	2020	9 (81.8%)	0.854	3 (27.3%)	0.755	2 (18.2%)	0.727	0 (0.0%)	**0.022**	2 (18.2%)	0.742
	2021	42 (87.5%)		17 (35.4%)		15 (31.3%)		22 (45.8%)		17 (35.4%)	
	2022	41 (82.0%)		13 (26.0%)		14 (28.0%)		17 (34.0%)		16 (32.0%)	
	2023	24 (82.8%)		10 (34.5%)		6 (20.7%)		9 (31.0%)		8 (27.6%)	
Clinical training	Pre-clinical Student	591 (90.4%)	0.604	218 (33.3%)	0.795	148 (22.6%)	0.304	259 (39.6%)	0.917	196 (30.0%)	0.086
	Clinical Student	1,174 (89.6%)		429 (32.7%)		324 (24.7%)		522 (39.8%)		443 (33.8%)	
Nationality	International Student	233 (91.0%)	0.514	86 (33.6%)	0.812	53 (20.7%)	0.181	93 (36.3%)	0.228	79 (30.9%)	0.539
	Domestic Student	1,532 (89.7%)		561 (32.8%)		419 (24.5%)		688 (40.3%)		560 (32.8%)	
Economic level	Low Income	59 (79.7%)	**<0.001**	23 (31.1%)	**<0.001**	25 (33.8%)	**0.045**	27 (36.5%)	0.081	31 (41.9%)	**0.001**
	Lower-middle Income	257 (83.4%)		112 (36.4%)		75 (24.4%)		107 (34.7%)		123 (39.9%)	
	Upper-middle Income	542 (86.4%)		256 (40.8%)		164 (26.2%)		242 (38.6%)		208 (33.2%)	
	High Income	907 (95.0%)		256 (26.8%)		208 (21.8%)		405 (42.4%)		277 (29.0%)	
Region	Africa	236 (83.1%)	**<0.001**	111 (39.1%)	**<0.001**	78 (27.5%)	0.221	102 (35.9%)	**0.030**	119 (41.9%)	**<0.001**
	Asia Pacific	152 (87.4%)		39 (22.4%)		35 (20.1%)		66 (37.9%)		63 (36.2%)	
	Europe	1,244 (91.7%)		434 (32.0%)		326 (24.0%)		550 (40.5%)		393 (29.0%)	
	South America	113 (88.3%)		58 (45.3%)		31 (24.2%)		60 (46.9%)		61 (47.7%)	
	North America	20 (95.2%)		5 (23.8%)		2 (9.5%)		3 (14.3%)		3 (14.3%)	
Financial aid	No Aid	1,368 (90.0%)	0.719	504 (33.2%)	0.708	357 (23.5%)	0.295	601 (39.5%)	0.705	476 (31.3%)	**0.033**
	Aid Received	397 (89.4%)		143 (32.2%)		115 (25.9%)		180 (40.5%)		163 (36.7%)	
Career planning training	Not Received	1,265 (91.1%)	**0.006**	428 (30.8%)	**0.002**	341 (24.6%)	0.404	550 (39.6%)	0.812	445 (32.0%)	0.464
	Received	500 (87.0%)		219 (38.1%)		131 (22.8%)		231 (40.2%)		194 (33.7%)	

Variable	Outcome	Agencies of career planning training				Preferred media of career planning training					
		Reg. Dent. Students Ass.	*p.*	Nat. Dent. Students Ass.	*p.*	Webinars	*p.*	Podcasts	*p.*	Blogs	*p.*
Gender	Female	389 (27.6%)	**0.030**	563 (39.9%)	0.109	733 (51.9%)	**0.020**	439 (31.1%)	0.098	377 (26.7%)	**<0.001**
	Male	126 (22.8%)		199 (36.0%)		255 (46.1%)		151 (27.3%)		104 (18.8%)	
Age group	≤22 years	297 (26.0%)	0.777	457 (40.0%)	0.204	532 (46.5%)	**<0.001**	341 (29.8%)	0.813	294 (25.7%)	0.134
	>22 years	218 (26.6%)		305 (37.1%)		456 (55.5%)		249 (30.3%)		187 (22.8%)	
Academic year	First year	80 (25.5%)	0.958	122 (38.9%)	0.989	130 (41.4%)	**<0.001**	105 (33.4%)	0.051	85 (27.1%)	0.684
	Second year	86 (25.3%)		132 (38.8%)		153 (45.0%)		95 (27.9%)		82 (24.1%)	
	Third year	102 (28.3%)		147 (40.7%)		180 (49.9%)		103 (28.5%)		90 (24.9%)	
	Fourth year	88 (24.3%)		142 (39.2%)		194 (53.6%)		119 (32.9%)		92 (25.4%)	
	Fifth year	81 (27.3%)		111 (37.4%)		157 (52.9%)		98 (33.0%)		69 (23.2%)	
	Sixth year	26 (25.7%)		39 (38.6%)		61 (60.4%)		23 (22.8%)		20 (19.8%)	
	Internship	14 (27.5%)		19 (37.3%)		28 (54.9%)		18 (35.3%)		15 (29.4%)	
	Graduated^╪^	38 (27.5%)		50 (36.2%)		85 (61.6%)		29 (21.0%)		28 (20.3%)	
^╪^Graduation year	2020	4 (36.4%)	0.717	6 (54.5%)	0.313	5 (45.5%)	0.112	2 (18.2%)	0.753	3 (27.3%)	0.468
	2021	15 (31.3%)		19 (39.6%)		35 (72.9%)		9 (18.8%)		10 (20.8%)	
	2022	12 (24.0%)		18 (36.0%)		31 (62.0%)		13 (26.0%)		12 (24.0%)	
	2023	7 (24.1%)		7 (24.1%)		14 (48.3%)		5 (17.2%)		3 (10.3%)	
Clinical training	Pre-clinical Student	166 (25.4%)	0.550	254 (38.8%)	0.980	283 (43.3%)	**<0.001**	200 (30.6%)	0.712	167 (25.5%)	0.447
	Clinical Student	349 (26.6%)		508 (38.8%)		705 (53.8%)		390 (29.8%)		314 (24.0%)	
Nationality	International Student	66 (25.8%)	0.863	89 (34.8%)	0.156	117 (45.7%)	0.114	63 (24.6%)	**0.042**	57 (22.3%)	0.375
	Domestic Student	449 (26.3%)		673 (39.4%)		871 (51.0%)		527 (30.9%)		424 (24.8%)	
Economic level	Low Income	24 (32.4%)	0.603	30 (40.5%)	0.126	36 (48.6%)	**0.002**	15 (20.3%)	0.061	15 (20.3%)	0.105
	Lower-middle Income	79 (25.6%)		111 (36.0%)		125 (40.6%)		92 (29.9%)		76 (24.7%)	
	Upper-middle Income	168 (26.8%)		226 (36.0%)		334 (53.3%)		174 (27.8%)		174 (27.8%)	
	High Income	244 (25.5%)		395 (41.4%)		493 (51.6%)		309 (32.4%)		216 (22.6%)	
Region	Africa	76 (26.8%)	0.849	113 (39.8%)	0.890	122 (43.0%)	**0.001**	81 (28.5%)	0.451	78 (27.5%)	0.284
	Asia Pacific	48 (27.6%)		68 (39.1%)		83 (47.7%)		48 (27.6%)		42 (24.1%)	
	Europe	350 (25.8%)		524 (38.6%)		693 (51.1%)		412 (30.4%)		320 (23.6%)	
	South America	37 (28.9%)		51 (39.8%)		82 (64.1%)		45 (35.2%)		38 (29.7%)	
	North America	4 (19.0%)		6 (28.6%)		8 (38.1%)		4 (19.0%)		3 (14.3%)	
Financial aid	No Aid	388 (25.5%)	0.195	585 (38.5%)	0.600	775 (51.0%)	0.264	448 (29.5%)	0.310	367 (24.1%)	0.509
	Aid Received	127 (28.6%)		177 (39.9%)		213 (48.0%)		142 (32.0%)		114 (25.7%)	
Career planning training	Not Received	373 (26.9%)	0.322	554 (39.9%)	0.125	713 (51.3%)	0.157	439 (31.6%)	**0.019**	349 (25.1%)	0.309
	Received	142 (24.7%)		208 (36.2%)		275 (47.8%)		151 (26.3%)		132 (23.0%)	

Variable	Outcome	Preferred media of career planning training									
		E-books	*p.*	CE Workshop	*p.*	UG course	*p.*				
Gender	Female	335 (23.7%)	0.980	869 (61.6%)	0.849	893 (63.3%)	0.602				
	Male	131 (23.7%)		338 (61.1%)		343 (62.0%)					
Age group	≤ 22 years	265 (23.2%)	0.505	653 (57.1%)	**<0.001**	727 (63.6%)	0.467				
	> 22 years	201 (24.5%)		554 (67.5%)		509 (62.0%)					
Academic year	First year	75 (23.9%)	0.904	171 (54.5%)	**0.002**	206 (65.6%)	0.707				
	Second year	71 (20.9%)		193 (56.8%)		216 (63.5%)					
	Third year	83 (23.0%)		218 (60.4%)		230 (63.7%)					
	Fourth year	89 (24.6%)		230 (63.5%)		222 (61.3%)					
	Fifth year	75 (25.3%)		191 (64.3%)		188 (63.3%)					
	Sixth year	26 (25.7%)		68 (67.3%)		64 (63.4%)					
	Internship	14 (27.5%)		35 (68.6%)		33 (64.7%)					
	Graduated^╪^	33 (23.9%)		101 (73.2%)		77 (55.8%)					
^╪^Graduation year	2020	1 (9.1%)	0.612	8 (72.7%)	0.762	5 (45.5%)	0.588				
	2021	11 (22.9%)		37 (77.1%)		27 (56.3%)					
	2022	14 (28.0%)		34 (68.0%)		31 (62.0%)					
	2023	7 (24.1%)		22 (75.9%)		14 (48.3%)					
Clinical training	Pre-clinical Student	146 (22.3%)	0.302	364 (55.7%)	**<0.001**	422 (64.5%)	0.302				
	Clinical Student	320 (24.4%)		843 (64.4%)		814 (62.1%)					
Nationality	International Student	63 (24.6%)	0.722	145 (56.6%)	0.090	161 (62.9%)	0.988				
	Domestic Student	403 (23.6%)		1,062 (62.2%)		1,075 (62.9%)					
Economic level	Low Income	21 (28.4%)	0.307	50 (67.6%)	0.521	38 (51.4%)	**0.040**				
	Lower-middle Income	73 (23.7%)		186 (60.4%)		189 (61.4%)					
	Upper-middle Income	161 (25.7%)		394 (62.8%)		383 (61.1%)					
	High Income	211 (22.1%)		577 (60.4%)		626 (65.5%)					
Region	Africa	75 (26.4%)	**0.045**	170 (59.9%)	**0.050**	180 (63.4%)	0.336				
	Asia Pacific	37 (21.3%)		105 (60.3%)		97 (55.7%)					
	Europe	307 (22.6%)		834 (61.5%)		861 (63.4%)					
	South America	43 (33.6%)		90 (70.3%)		84 (65.6%)					
	North America	4 (19.0%)		8 (38.1%)		14 (66.7%)					
Financial aid	No aid	351 (23.1%)	0.221	952 (62.6%)	**0.048**	958 (63.0%)	0.874				
	Aid received	115 (25.9%)		255 (57.4%)		278 (62.6%)					
Career planning training	Not received	304 (21.9%)	**0.003**	874 (62.9%)	**0.038**	875 (63.0%)	0.929				
	Received	162 (28.2%)		333 (57.9%)		361 (62.8%)					

Chi-squared (*χ^2^*) and Fisher's exact tests were used with a significance level (*p*.) < 0.05.

Bold font refers to the values that are statistically significant.

The most frequently suggested medium for providing career planning training was undergraduate elective courses (62.9%), followed closely by continuous education workshops (61.5%). Virtual media were less frequently suggested: webinars (50.3%), podcasts (30%), blogs (24.5%), and e-books (23.7%). Regarding the timing of undergraduate courses, less than half (49.5%) recommended that these courses be provided during the first years of dental school, while 34.5% suggested they be offered towards graduation during the senior years, and 16% recommended the middle years of dental school, typically coinciding with the beginning of clinical training. Among recent graduates, 84.7% believed that continuous education programs should include components on career management (Table 4).

Female students were significantly more in favour of webinars (51.9% vs. 46.1%; *p.* = 0.020) and blogs (26.7% vs. 18.8%; *p.* < 0.001). Students aged over 22 years were more likely to favour webinars (55.5% vs. 46.5%; *p.* < 0.001) and continuous education workshops (67.5% vs. 57.1%; *p.* < 0.001). Clinical students were more inclined to favour webinars (53.8% vs. 43.3%; *p.* < 0.001) and continuous education workshops (64.4% vs. 55.7%; *p.* < 0.001). Domestic students were more likely to favour podcasts (30.9% vs. 24.6%; *p.* = 0.042) (Table 6).

Students aged over 22 years were also more likely to support the provision of undergraduate career planning courses in the senior years (24.6% vs. 9.8%; *p.* < 0.001). Similarly, clinical students were more supportive of offering these courses in the senior years (21.1% vs. 5.8%; *p.* < 0.001). International students reported receiving career planning training more frequently than their domestic counterparts (36.3% vs. 28.2%; *p.* = 0.008), and this was also more common among those who received financial aid compared to those who did not (34.2% vs. 27.8%; *p.* = 0.009) (Table 7).

**Table 7:** Receiving career planning training and its preferred timing in undergraduate curriculum as reported by dental students participating in FDI–IADS–EDSA survey of career planning, stratified by sociodemographic characteristics, May – July 2023, (*n* = 1,964).

Variable	Outcome	Received career planning training?			Preferred timing of career planning training in dental school			
		No	Yes	*p.*	Upon entry (freshman years)	Beginning of clinical years (e.g., 3rd, 4th Yr.)	Towards graduation (senior years)	*p.*
Gender	Female	1,002 (71.0%)	409 (29.0%)	0.651	502 (35.6%)	698 (49.5%)	211 (15.0%)	0.083
	Male	387 (70.0%)	166 (30.0%)		176 (31.8%)	274 (49.5%)	103 (18.6%)	
Age group	≤22 years	802 (70.2%)	341 (29.8%)	0.522	484 (42.3%)	547 (47.9%)	112 (9.8%)	**<0.001**
	>22 years	587 (71.5%)	234 (28.5%)		194 (23.6%)	425 (51.8%)	202 (24.6%)	
Academic year	First year	201 (64.0%)	113 (36.0%)	**0.006**	171 (54.5%)	124 (39.5%)	19 (6.1%)	**<0.001**
	Second year	233 (68.5%)	107 (31.5%)		148 (43.5%)	173 (50.9%)	19 (5.6%)	
	Third year	278 (77.0%)	83 (23.0%)		123 (34.1%)	198 (54.8%)	40 (11.1%)	
	Fourth year	273 (75.4%)	89 (24.6%)		107 (29.6%)	185 (51.1%)	70 (19.3%)	
	Fifth year	204 (68.7%)	93 (31.3%)		72 (24.2%)	151 (50.8%)	74 (24.9%)	
	Sixth year	71 (70.3%)	30 (29.7%)		22 (21.8%)	56 (55.4%)	23 (22.8%)	
	Internship	33 (64.7%)	18 (35.3%)		8 (15.7%)	25 (49.0%)	18 (35.3%)	
	Graduated^╪^	96 (69.6%)	42 (30.4%)		27 (19.6%)	60 (43.5%)	51 (37.0%)	
^╪^Graduation year	2020	5 (45.5%)	6 (54.5%)	0.317	3 (27.3%)	6 (54.5%)	2 (18.2%)	0.648
	2021	34 (70.8%)	14 (29.2%)		6 (12.5%)	22 (45.8%)	20 (41.7%)	
	2022	37 (74.0%)	13 (26.0%)		12 (24.0%)	19 (38.0%)	19 (38.0%)	
	2023	20 (69.0%)	9 (31.0%)		6 (20.7%)	13 (44.8%)	10 (34.5%)	
Clinical training	Pre-clinical Student	434 (66.4%)	220 (33.6%)	**0.003**	319 (48.8%)	297 (45.4%)	38 (5.8%)	**<0.001**
	Clinical Student	955 (72.9%)	355 (27.1%)		359 (27.4%)	675 (51.5%)	276 (21.1%)	
Nationality	International Student	163 (63.7%)	93 (36.3%)	**0.008**	91 (35.5%)	128 (50.0%)	37 (14.5%)	0.764
	Domestic Student	1,226 (71.8%)	482 (28.2%)		587 (34.4%)	844 (49.4%)	277 (16.2%)	
Economic level	Low Income	49 (66.2%)	25 (33.8%)	**0.009**	22 (29.7%)	34 (45.9%)	18 (24.3%)	0.337
	Lower-middle Income	224 (72.7%)	84 (27.3%)		103 (33.4%)	156 (50.6%)	49 (15.9%)	
	Upper-middle Income	414 (66.0%)	213 (34.0%)		212 (33.8%)	306 (48.8%)	109 (17.4%)	
	High Income	702 (73.5%)	253 (26.5%)		341 (35.7%)	476 (49.8%)	138 (14.5%)	
Region	Africa	200 (70.4%)	84 (29.6%)	0.472	101 (35.6%)	139 (48.9%)	44 (15.5%)	0.057
	Asia Pacific	122 (70.1%)	52 (29.9%)		55 (31.6%)	81 (46.6%)	38 (21.8%)	
	Europe	964 (71.0%)	393 (29.0%)		483 (35.6%)	672 (49.5%)	202 (14.9%)	
	South America	85 (66.4%)	43 (33.6%)		33 (25.8%)	72 (56.3%)	23 (18.0%)	
	North America	18 (85.7%)	3 (14.3%)		6 (28.6%)	8 (38.1%)	7 (33.3%)	
Financial aid	No Aid	1,097 (72.2%)	423 (27.8%)	**0.009**	499 (32.8%)	772 (50.8%)	249 (16.4%)	**0.014**
	Aid Received	292 (65.8%)	152 (34.2%)		179 (40.3%)	200 (45.0%)	65 (14.6%)	

Chi-squared (*χ^2^*) and Fisher's exact tests were used with a significance level (*p*.) < 0.05.

Bold font refers to the values that are statistically significant.

### Multivariable logistic regression analyses

To account for the confounding effects of various sociodemographic and psychological factors, multivariable logistic regression (MLR) models were built to identify predictors of career path preferences, suggested agencies for career planning, and preferred media for career planning training.

The decision to choose general dentistry as a career path was promoted by age [AOR: 1.07 (95% CI: 1.03–1.13)] and personal expectations [1.04 (1.01–1.08)]. Compared to students from low-income countries, those from lower-middle-income countries [2.18 (1.05–4.51)], upper-middle-income countries [1.37 (0.46–4.06)], and high-income countries [2.12 (0.72–6.27)] were more likely to prefer general dentistry. On the contrary, specialty dentistry was dissuaded by age [0.95 (0.91–0.99)] and personal expectations [0.96 (0.93–0.99)]. Compared to students from low-income countries, those from lower-middle-income countries [0.44 (0.25–0.76)], upper-middle-income countries [0.37 (0.16–0.84)], and high-income countries [0.30 (0.13–0.68)] were less likely to prefer specialty dentistry. Professional expectations [1.04 (1.01–1.06)] and career goals [1.03 (1.00–1.06)] were psychological promoters of specialty dentistry (Table 8).

**Table 8:** Multivariable logistic regression (MLR) models of first career preferences, suggested agencies, and preferred Media as reported by dental students participating in FDI–IADS–EDSA survey of career planning, May – July 2023, (*n* = 1,964).

Predictor	Preferred career pathways									
	General dentistry		Specialty dentistry		Business		Academia		Public health	
	AOR (95% CI)	*p*.	AOR (95% CI)	*p*.	AOR (95% CI)	*p*.	AOR (95% CI)	*p*.	AOR (95% CI)	*p*.
Gender (Male vs. Female)	0.92 (0.73–1.16)	0.484	0.94 (0.77–1.15)	0.547	2.44 (1.55–3.84)	**<0.001**	0.97 (0.56–1.68)	0.925	1.44 (0.75–2.76)	0.279
Age (continuous)	1.07 (1.03–1.13)	**0.002**	0.95 (0.91–0.99)	**0.021**	1.02 (0.92–1.13)	0.740	0.94 (0.81–1.10)	0.438	1.14 (1.00–1.28)	**0.042**
Academic Year (continuous)	1.04 (0.97–1.11)	0.296	1.03 (0.97–1.10)	0.337	0.93 (0.79–1.08)	0.334	1.11 (0.92–1.34)	0.293	0.82 (0.68–1.00)	**0.049**
Nationality (Inter vs. Dome)	1.02 (0.76–1.38)	0.892	0.85 (0.65–1.12)	0.253	1.10 (0.56–2.15)	0.792	2.89 (0.89–9.45)	0.079	2.06 (0.48–8.89)	0.333
SDI (Lower-mid. vs. Low)	2.18 (1.05–4.51)	**0.036**	0.44 (0.25–0.76)	**0.003**	1.62 (0.34–7.73)	0.546	11.19 (1.45–86.61)	**0.021**	0.23 (0.08–0.66)	**0.006**
SDI (Upper-mid. vs. Low)	1.37 (0.46–4.06)	0.574	0.37 (0.16–0.84)	**0.018**	4.38 (0.41–46.66)	0.221	4.77 (0.34–66.39)	0.245	0.32 (0.05–2.14)	0.239
SDI (High vs. Low)	2.12 (0.72–6.27)	0.175	0.30 (0.13–0.68)	**0.004**	3.38 (0.32–36.23)	0.315	2.67 (0.19–37.71)	0.466	0.18 (0.02–1.26)	0.084
Region (Asia vs. Africa)	0.67 (0.37–1.23)	0.194	2.17 (1.31–3.58)	**0.002**	0.45 (0.10–2.03)	0.300	0.51 (0.20–1.33)	0.170	1.29 (0.37–4.53)	0.693
Region (Europe vs. Africa)	1.68 (0.71–3.98)	0.243	1.84 (0.93–3.63)	0.080	0.44 (0.07–2.90)	0.393	0.69 (0.13–3.83)	0.673	0.25 (0.04–1.50)	0.131
Region (S. America vs. Africa)	0.60 (0.22–1.68)	0.334	3.77 (1.70–8.40)	**0.001**	0.24 (0.02–2.23)	0.207	0.48 (0.06–3.94)	0.491	0.77 (0.10–5.89)	0.803
Region (N. America vs. Africa)	3.45 (1–11.95)	0.051	1.19 (0.39–3.65)	0.755	0.40 (0.02–6.40)	0.516	0.81 (0.06–11.71)	0.879	0.99 (0.07–14.24)	0.992
Financial Aid (Yes vs. No)	1.07 (0.84–1.36)	0.593	0.93 (0.75–1.15)	0.502	1.15 (0.68–1.94)	0.614	2.12 (1.24–3.61)	**0.006**	1.08 (0.51–2.32)	0.835
CP Training (Received vs. No)	1.17 (0.93–1.47)	0.188	0.95 (0.77–1.16)	0.593	1.07 (0.64–1.79)	0.801	0.80 (0.45–1.41)	0.432	0.51 (0.22–1.15)	0.104
Self-efficacy	1.02 (0.99–1.05)	0.274	1.02 (0.99–1.05)	0.161	0.98 (0.91–1.04)	0.485	1.01 (0.94–1.09)	0.771	0.96 (0.87–1.04)	0.311
Professional Expectations	0.98 (0.96–1.01)	0.191	1.04 (1.01–1.06)	**0.005**	0.97 (0.91–1.02)	0.212	0.98 (0.92–1.05)	0.542	0.97 (0.90–1.05)	0.407
Personal Expectations	1.04 (1.01–1.08)	**0.018**	0.96 (0.93–0.99)	**0.004**	0.96 (0.89–1.03)	0.285	1.01 (0.93–1.09)	0.896	1.02 (0.92–1.13)	0.745
Career Goals	0.99 (0.96–1.02)	0.501	1.03 (1.00–1.06)	**0.036**	0.99 (0.93–1.06)	0.864	1.03 (0.96–1.11)	0.410	1.01 (0.92–1.10)	0.888

Predictor	Agencies of career planning training									
	Dental schools		FDI		Regional Dent. Ass.		National Dent. Ass.		IADS	
	AOR (95% CI)	*p*.	AOR (95% CI)	*p*.	AOR (95% CI)	*p*.	AOR (95% CI)	*p*.	AOR (95% CI)	*p*.
Gender (Male vs. Female)	0.84 (0.61–1.17)	0.298	0.81 (0.65–1.01)	0.057	0.86 (0.68–1.09)	0.208	1.26 (1.03–1.55)	**0.028**	0.70 (0.56–0.88)	**0.002**
Age (continuous)	0.98 (0.91–1.05)	0.544	0.96 (0.91–1.00)	0.067	1.01 (0.96–1.06)	0.698	1.00 (0.96–1.05)	0.996	0.97 (0.92–1.01)	0.147
Academic Year (continuous)	1.06 (0.95–1.18)	0.287	1.03 (0.97–1.11)	0.339	0.98 (0.91–1.06)	0.659	0.97 (0.91–1.04)	0.363	1.02 (0.95–1.09)	0.632
Nationality (Inter vs. Dome)	1.03 (0.64–1.66)	0.905	0.85 (0.63–1.14)	0.267	1.19 (0.85–1.66)	0.302	1.21 (0.91–1.61)	0.186	0.88 (0.65–1.19)	0.403
SDI (Lower-mid. vs. Low)	1.48 (0.73–3.00)	0.273	1.59 (0.89–2.84)	0.115	0.66 (0.36–1.18)	0.161	0.91 (0.52–1.59)	0.734	0.99 (0.57–1.72)	0.960
SDI (Upper-mid. vs. Low)	3.67 (0.95–14.14)	0.059	1.50 (0.59–3.81)	0.400	0.51 (0.19–1.34)	0.171	1.27 (0.54–2.96)	0.583	0.88 (0.38–2.05)	0.764
SDI (High vs. Low)	11.20 (2.82–44.39)	**<0.001**	0.83 (0.33–2.14)	0.706	0.38 (0.14–0.99)	**0.048**	1.58 (0.68–3.69)	0.289	0.88 (0.38–2.06)	0.775
Region (Asia vs. Africa)	0.78 (0.40–1.51)	0.459	0.45 (0.26–0.77)	**0.004**	0.89 (0.50–1.58)	0.683	1.02 (0.61–1.70)	0.954	0.81 (0.49–1.35)	0.416
Region (Europe vs. Africa)	0.44 (0.13–1.50)	0.189	1.01 (0.46–2.23)	0.974	1.47 (0.64–3.37)	0.368	0.81 (0.41–1.62)	0.549	0.62 (0.31–1.24)	0.172
Region (S. America vs. Africa)	0.51 (0.13–1.98)	0.329	1.46 (0.60–3.52)	0.401	1.24 (0.48–3.20)	0.660	1.29 (0.58–2.86)	0.531	1.79 (0.80–4.02)	0.157
Region (N. America vs. Africa)	1.17 (0.11–12.30)	0.899	0.52 (0.14–1.87)	0.315	0.42 (0.08–2.25)	0.310	0.22 (0.05–0.90)	**0.035**	0.27 (0.07–1.12)	0.071
Financial Aid (Yes vs. No)	0.93 (0.65–1.33)	0.688	0.99 (0.79–1.25)	0.959	1.16 (0.90–1.48)	0.243	1.04 (0.84–1.30)	0.702	1.35 (1.08–1.70)	**0.009**
CP Training (Received vs. No)	0.67 (0.48–0.93)	**0.016**	1.35 (1.09–1.68)	**0.007**	0.88 (0.69–1.13)	0.319	1.11 (0.90–1.37)	0.334	1.08 (0.87–1.35)	0.486
Self-efficacy	0.97 (0.93–1.02)	0.191	0.99 (0.96–1.02)	0.516	1.01 (0.98–1.04)	0.644	0.98 (0.96–1.01)	0.226	0.99 (0.97–1.02)	0.677
Professional Expectations	1.04 (1.00–1.09)	**0.039**	1.02 (0.99–1.04)	0.227	1.02 (0.99–1.05)	0.127	1.05 (1.02–1.07)	**<0.001**	1.05 (1.02–1.08)	**<0.001**
Personal Expectations	1.03 (0.98–1.08)	0.266	0.97 (0.94–1.00)	**0.050**	0.97 (0.93–1.00)	0.082	0.97 (0.94–1.00)	**0.032**	0.93 (0.90–0.96)	**<0.001**
Career Goals	1.00 (0.95–1.04)	0.871	1.01 (0.98–1.04)	0.594	0.99 (0.96–1.02)	0.426	0.96 (0.94–0.99)	**0.008**	0.99 (0.96–1.02)	0.353

Predictor	Agencies of career planning training				Preferred media of career planning training					
	Regional Dent. Stu. Ass.		National Dent. Stu. Ass.		Webinars		Podcasts		Blogs	
	AOR (95% CI)	*p*.	AOR (95% CI)	*p*.	AOR (95% CI)	*p*.	AOR (95% CI)	*p*.	AOR (95% CI)	*p*.
Gender (Male vs. Female)	0.78 (0.62–0.99)	**0.041**	0.90 (0.73–1.10)	0.305	0.77 (0.62–0.94)	**0.010**	0.86 (0.69–1.07)	0.182	0.66 (0.51–0.84)	**<0.001**
Age (continuous)	1.01 (0.96–1.06)	0.709	0.99 (0.94–1.03)	0.582	1.04 (0.99–1.09)	0.088	1.01 (0.97–1.06)	0.630	0.97 (0.92–1.03)	0.295
Academic Year (continuous)	1.00 (0.93–1.07)	0.909	1.00 (0.94–1.06)	0.960	1.10 (1.04–1.18)	**0.002**	0.96 (0.90–1.03)	0.235	1.00 (0.92–1.07)	0.905
Nationality (Inter vs. Dome)	0.95 (0.70–1.30)	0.760	1.19 (0.89–1.58)	0.244	1.14 (0.86–1.50)	0.354	1.39 (1.02–1.90)	**0.039**	1.04 (0.75–1.44)	0.823
SDI (Lower-mid. vs. Low)	0.70 (0.39–1.27)	0.246	0.93 (0.53–1.62)	0.796	0.89 (0.51–1.54)	0.666	1.74 (0.91–3.33)	0.094	1.66 (0.86–3.19)	0.128
SDI (Upper-mid. vs. Low)	0.66 (0.27–1.63)	0.368	2.00 (0.86–4.67)	0.109	2.04 (0.88–4.70)	0.095	1.51 (0.59–3.87)	0.392	6.49 (2.41–17.48)	**<0.001**
SDI (High vs. Low)	0.61 (0.25–1.50)	0.280	2.63 (1.13–6.13)	**0.026**	2.03 (0.88–4.69)	0.096	1.95 (0.76–5.00)	0.162	5.04 (1.87–13.58)	**0.001**
Region (Asia vs. Africa)	1.20 (0.69–2.09)	0.517	0.66 (0.39–1.12)	0.123	0.76 (0.46–1.26)	0.293	0.83 (0.48–1.43)	0.498	0.38 (0.20–0.73)	**0.004**
Region (Europe vs. Africa)	1.16 (0.55–2.46)	0.698	0.38 (0.19–0.75)	**0.006**	0.65 (0.33–1.29)	0.219	0.91 (0.43–1.90)	0.799	0.22 (0.10–0.48)	**<0.001**
Region (S. America vs. Africa)	1.30 (0.54–3.10)	0.556	0.49 (0.22–1.09)	0.079	0.84 (0.38–1.85)	0.657	1.34 (0.57–3.13)	0.501	0.28 (0.11–0.70)	**0.006**
Region (N. America vs. Africa)	0.76 (0.20–2.88)	0.688	0.26 (0.08–0.86)	**0.026**	0.32 (0.10–1.00)	0.051	0.54 (0.14–2.02)	0.357	0.11 (0.02–0.46)	**0.003**
Financial Aid (Yes vs. No)	1.17 (0.92–1.49)	0.194	1.05 (0.84–1.31)	0.691	0.87 (0.70–1.08)	0.202	1.18 (0.93–1.49)	0.169	1.09 (0.85–1.40)	0.489
CP Training (Received vs. No)	0.90 (0.71–1.14)	0.384	0.86 (0.69–1.06)	0.157	0.90 (0.73–1.10)	0.303	0.80 (0.63–1.00)	0.053	0.84 (0.66–1.07)	0.157
Self-efficacy	0.99 (0.96–1.02)	0.651	0.98 (0.96–1.01)	0.192	0.97 (0.95–1.00)	0.053	0.97 (0.94–1.00)	**0.032**	1.01 (0.98–1.04)	0.481
Professional Expectations	1.04 (1.02–1.07)	**0.003**	1.05 (1.02–1.07)	**<0.001**	1.02 (0.99–1.04)	0.166	1.01 (0.99–1.04)	0.354	1.01 (0.98–1.04)	0.377
Personal Expectations	0.98 (0.95–1.02)	0.281	0.98 (0.95–1.01)	0.132	1.00 (0.97–1.03)	0.994	1.01 (0.97–1.04)	0.631	1.00 (0.96–1.04)	0.909
Career Goals	0.97 (0.94–1.00)	**0.048**	1.00 (0.97–1.02)	0.732	1.00 (0.97–1.03)	0.906	1.01 (0.98–1.04)	0.417	0.99 (0.96–1.02)	0.409
Predictor	Preferred media of career planning training									
	E-books		CE workshop		UG course					
	AOR (95% CI)	*p*.	AOR (95% CI)	*p*.	AOR (95% CI)	*p*.				
Gender (Male vs. Female)	0.98 (0.77–1.24)	0.852	0.94 (0.76–1.16)	0.556	0.96 (0.78–1.18)	0.693				
Age (continuous)	0.99 (0.94–1.04)	0.796	1.07 (1.02–1.12)	**0.006**	1.00 (0.96–1.05)	0.847				
Academic Year (continuous)	1.03 (0.96–1.11)	0.430	1.05 (0.98–1.12)	0.142	0.98 (0.92–1.04)	0.507				
Nationality (Inter vs. Dome)	0.90 (0.66–1.24)	0.532	1.19 (0.90–1.57)	0.228	1.03 (0.78–1.37)	0.818				
SDI (Lower-mid. vs. Low)	0.95 (0.52–1.75)	0.877	0.71 (0.40–1.27)	0.250	1.92 (1.11–3.33)	**0.020**				
SDI (Upper-mid. vs. Low)	1.18 (0.45–3.07)	0.736	0.42 (0.18–0.99)	**0.048**	3.26 (1.41–7.55)	**0.006**				
SDI (High vs. Low)	1.09 (0.42–2.83)	0.867	0.38 (0.16–0.88)	**0.025**	4.15 (1.79–9.62)	**<0.001**				
Region (Asia vs. Africa)	0.67 (0.37–1.22)	0.189	1.35 (0.81–2.26)	0.249	0.48 (0.29–0.79)	**0.004**				
Region (Europe vs. Africa)	0.69 (0.31–1.53)	0.360	2.07 (1.04–4.13)	**0.038**	0.42 (0.21–0.84)	**0.014**				
Region (S. America vs. Africa)	1.09 (0.44–2.70)	0.851	2.03 (0.90–4.55)	0.087	0.55 (0.24–1.23)	0.142				
Region (N. America vs. Africa)	0.53 (0.14–2.04)	0.352	0.62 (0.20–1.93)	0.412	0.57 (0.18–1.80)	0.339				
Financial Aid (Yes vs. No)	1.16 (0.90–1.48)	0.245	0.81 (0.65–1.01)	0.064	0.99 (0.79–1.24)	0.935				
CP Training (Received vs. No)	1.34 (1.06–1.69)	**0.015**	0.79 (0.64–0.97)	**0.028**	1.02 (0.83–1.26)	0.846				
Self-efficacy	0.99 (0.96–1.02)	0.387	0.98 (0.96–1.01)	0.220	1.00 (0.97–1.02)	0.787				
Professional Expectations	1.01 (0.98–1.04)	0.382	1.04 (1.01–1.07)	**0.002**	1.03 (1.00–1.05)	**0.042**				
Personal Expectations	1.01 (0.97–1.05)	0.657	0.98 (0.95–1.01)	0.184	0.99 (0.96–1.02)	0.465				
Career Goals	1.01 (0.98–1.04)	0.510	1.01 (0.98–1.04)	0.453	0.98 (0.95–1.01)	0.181				

Bold font refers to the values that are statistically significant.

The only significant promoter of a business/entrepreneurship career path was male gender [2.44 (1.55–3.84)]. Receiving financial aid was a significant promoter of an academic career path [2.12 (1.24–3.61)], as was being from lower-middle-income countries [11.19 (1.45–86.61)]. The public health career path was promoted by age [1.14 (1.00–1.28)], and it was less common among students from lower-middle-income countries [0.23 (0.08–0.66)], upper-middle-income countries [0.32 (0.05–2.14)], and high-income countries [0.18 (0.02–1.26)] as compared with those from low-income countries (Table 8).

The perceived competency of dental schools in providing career planning training was positively associated with higher professional expectations [1.04 (1.00–1.09)] and being from high-income countries [11.20 (2.82–44.39)], but negatively associated with prior career planning training [0.67 (0.48–0.93)]. In contrast, prior training increased the likelihood of suggesting the FDI as a competent agency [1.35 (1.09–1.68)]. Regional dental associations were less favoured by students from high-income countries [0.38 (0.14–0.99)], upper-middle-income countries [0.51 (0.19–1.34)], and lower-middle-income countries [0.66 (0.36–1.18)] compared to those from low-income countries. Professional expectations [1.05 (1.02–1.07)] and male gender [1.26 (1.03–1.55)] were positively associated with higher odds of favouring national dental associations (Table 8).

The IADS was less commonly preferred by male students [0.70 (0.56–0.88)], whereas it was more commonly preferred by students who received financial aid [1.35 (1.08–1.70)]. Higher professional expectations [1.05 (1.02–1.08)] increased the odds of favouring the IADS, while higher personal expectations [0.93 (0.90–0.96)] decreased them. Additionally, male gender was associated with lower odds of favouring regional dental students' associations, such as EDSA [0.78 (0.62–0.99)] (Table 8).

Virtual media outlets were less favoured by male students, including webinars [0.77 (0.62–0.94)] and blogs [0.66 (0.51–0.84)]. Advancing age and academic year were associated with higher odds of favouring continuous education workshops [1.07 (1.02–1.12)] and webinars [1.10 (1.04–1.18)], respectively. Compared to students from low-income countries, continuous education workshops were less preferred by students from lower-middle-income [0.71 (0.40–1.27)], upper-middle-income [0.42 (0.18–0.99)], and high-income countries [0.38 (0.16–0.88)]. Conversely, undergraduate courses were more preferred by students from lower-middle-income [1.92 (1.11–3.33)], upper-middle-income [3.26 (1.41–7.55)], and high-income countries [4.15 (1.79–9.62)] (Table 8).

### Mediation analyses

The mediation analyses aimed to explore the effect of prior career planning training on preferred career paths through the SCCT components.

**Figure 2:**
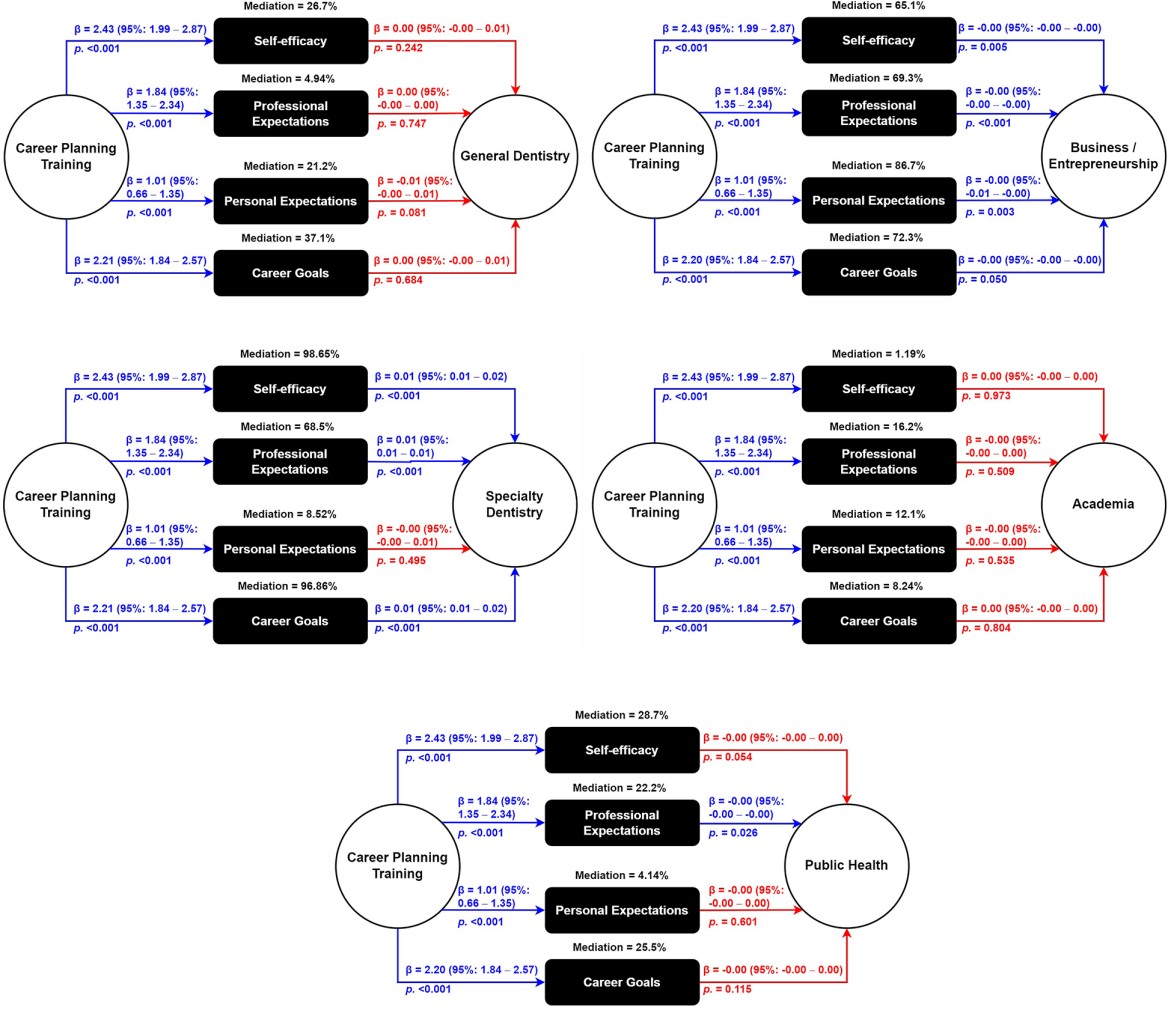
Mediation analysis for the effect of self-efficacy, professional expectations, personal expectations, and career goals on the relationship between career planning training (predictor) and primary career preferences (outcome) among dental students participating in the FDI–IADS–EDSA survey of career planning, May–July 2023, (*n* = 1,964).

For general dentistry, career planning training significantly enhanced self-efficacy [2.43 (1.99–2.87)], professional expectations [1.84 (1.35–2.34)], personal expectations [1.01 (0.66–1.35)], and career goals [2.21 (1.84–2.57)]. However, none of these factors had a significant direct effect on the preference for general dentistry, indicating that these factors partially mediated the relationship between career planning training and the choice of general dentistry with mediation contributions of 26.7%, 4.94%, 21.2%, and 37.1% respectively (Figure 2A).

For specialty dentistry, training significantly increased self-efficacy [2.43 (1.99–2.87)], professional expectations [1.84 (1.35–2.34)], personal expectations [1.01 (0.66–1.35)], and career goals [2.21 (1.84–2.57)]. Self-efficacy [0.01 (0.01–0.02)], professional expectations [0.01 (0.01–0.01)], and career goals [0.01 (0.01–0.02)] had significant direct effects on specialty dentistry preference, demonstrating substantial mediation with contributions of 98.65%, 68.5%, and 96.86%, respectively (Figure 2B).

For business/entrepreneurship, training improved self-efficacy [2.43 (1.99–2.87)], professional expectations [1.84 (1.35–2.34)], personal expectations [1.01 (0.66–1.35)], and career goals [2.20 (1.84–2.57)]. However, all these factors had direct negative effects on this career path, with considerable mediation contributions of 65.1%, 69.3%, 86.7%, and 72.3% (Figure 2C).

For academia, training enhanced self-efficacy [2.43 (1.99–2.87)], professional expectations [1.84 (1.35–2.34)], personal expectations [1.01 (0.66–1.35)], and career goals [2.20 (1.84–2.57)]. None of these factors had a significant direct impact on preferring academia, indicating minimal or negligible mediation with contributions of 1.19%, 16.2%, 12.1%, and 8.24% (Figure 2D).

For public health, training increased self-efficacy [2.43 (1.99–2.87)], professional expectations [1.84 (1.35–2.34)], personal expectations [1.01 (0.66–1.35)], and career goals [2.20 (1.84–2.57)]. Nevertheless, all these factors negatively influenced the preference for public health, with partial mediation contributions of 28.7%, 22.2%, 4.14% and 25.5% (Figure 2E).

## Discussion

This study aimed to explore how aspects of the SCCT, such as self-efficacy, outcome expectations and career goals, shape the career decisions of dental students across various career pathways such as General Dentistry, Specialty Dentistry, Business/Entrepreneurship, Academia and Public Health. Expectations and influences in students' career choices have long been a topic of interest as such insights help shape the approach of educational institutions in providing guidance during the students' formational years (1, 11). Previous studies have explored factors such as professional satisfaction, intrinsic motivations and financial stability (20–28).

To the best of our knowledge, the currently available literature is mostly region-specific and does not implement a comprehensive theoretical model in their analysis. For instance, Gallagher et al. (2008, 2009) examined career expectations among dental students at a single London Dental School (20, 21). Ellakany et al. (2023) focused on career satisfaction among dental students and dentists in Saudi Arabia, while Che Musa et al. (2016) analysed career influences among dental students in Malaysia, each with a narrow national focus (22, 23). Additionally, Sam et al. (2016) investigated working environments and speciality choices among students in a single university in Saudi Arabia, with a similar narrow focus and lack of comprehensive modelling (29). These studies illustrate the regional focus of current literature and, while diverse in their investigated aspects, they lack a holistic theoretical approach, thus limiting their broader applicability. In this aspect, the present study is a pioneer in providing a more nuanced understanding, through the prism of the SCCT framework, into the influencing factors of career path choices of dental students globally, addressing the gaps in regionally focused studies. Given the nature of the study design, the findings provide crucial insight for developing targeted interventions and career guidance programs to support dental students' decision-making process in their career planning, tailored to the specific needs of their diverse environmental context.

### Self-efficacy in career planning

The findings revealed levels of uncertainty among students regarding their capacity to make an informed decision on their career pathway such that it would align with their goals, personality and lifestyle. It is important to explore this uncertainty within the context of the student's education as much as their environment. The multivariable regression analysis showed that students who reported career planning training (CPT) exhibited a higher self-efficacy, highlighting the importance of structured guidance in boosting their confidence in the decision-making process. This aligns accurately with existing literature and additionally with the mediation analysis, which highlighted CPT as a key mediator, positively impacting self-efficacy, especially for those pursuing speciality dentistry (28, 30, 31). When placed within the sociodemographic context, the higher self-efficacy among international and pre-clinical students could indicate that early career exploration and early exposure to information on the diverse opportunities within their field may help students feel more confident about their career choices (25, 27).

Another interesting finding was the difference in self-efficacy between pre-clinical and clinical students, with the former reporting higher levels of self-efficacy. The drop in self-efficacy seen in clinical students may be attributed to increased pressures and challenges they face during their training, as they are exposed to the demands of clinical practice and the complexities of patient care (32, 33).

### Outcome expectations

Notably high professional expectations among participants were observed, with over 80% expecting intellectual stimulation and work satisfaction, in accordance with the outcomes of Gallagher et al. (20, 21)

CPT is observed, again, as a significant factor in boosting both professional and personal expectations, with students who received prior training on their career planning reporting higher confidence in their future careers. The mediation analysis revealed that professional expectations served as a significant mediator in the preference for speciality dentistry, where students who received CPT exhibited a stronger likelihood of pursuing advanced specialities. These findings reinforce the role of structured guidance in shaping professional expectations and satisfaction (21–23).

International students and those in their pre-clinical years showing higher professional expectations as compared to domestic and clinical students is a reiteration of the importance of early exposure to more diverse healthcare system settings and career opportunities as well as the need for ongoing career support through the senior years of their studies, to tackle the gap in perception between students' early expectations and the realities they encounter later into their career (30, 33).

Personal expectations—such as work-life balance, lifestyle compatibility and participation in leisure activities—were also influenced by CPT. The regression analysis revealed that students with higher personal expectations were more likely to choose general dentistry. This could be attributed to lifestyle factors weighing in on the decision to pursue a less demanding and stable career path. The mediation analysis did not show personal expectations as a strong mediator, especially in terms of special dentistry, as compared to professional expectations; however, they still played a role in shaping career preferences.

Overall, personal and professional expectations were significant mediators across all career paths, showing that CPT helps clarify students' understanding of how their careers can meet personal and professional needs.

### Career goals and preferences

A significantly high portion of the participants reported having clear career goals (62.5%) and discussing their goals with their families and partners (73.7%) This finding places the role of community and social support in a strongly shaping position for students' career aspirations (34).

Another interesting factor playing into the career goal clarity was found to be financial aid. The findings suggest that economic support can alleviate the pressure of financial burden and allow students to focus more on their long-term professional development (25). This aligns with other studies where financial concerns were evaluated to dictate or limit students' career choices, particularly in lower-income regions (28, 32, 35).

Consistent with goal-setting theory, students with well-defined career goals were more likely to pursue specialty careers and entrepreneurial paths (36). The multivariable logistic regression analysis identified age and personal expectations as key predictors for career path preferences.

The mediation analysis further demonstrated that career planning training (CPT) positively influenced self-efficacy, professional expectations, and career goals, especially for students choosing specialty dentistry. In concordance with current literature, the dominance of specialty clinical dentistry (51.2%) as the most preferred career path, both as a first and second choice, indicates a strong interest among dental students to specialize, particularly in lower-income countries (23, 25, 28). General clinical dentistry on the other hand was more preferred among older students and those in their clinical years and higher-income countries. This could be attributed to the desire for faster integration into the workforce and job stability among senior dental students or recent graduates, which is consistent with previous findings from Gallagher *et al*. (20, 21) Similarly, such preference from students in higher-income countries could also be attributed to the perceived financial stability and job market within the country's context (30–32).

However, according to the mediation analysis results, for non-clinical paths such as business/entrepreneurship and public health, CPT seemed to negatively influence preferences. Public health was less popular among students from higher-income countries, which was unexpected. While previous research has suggested public health careers might appeal to students due to their societal impact, the results indicate that financial considerations may deter students from pursuing public health, especially in wealthier regions (20, 21). Conversely, students from lower-income regions demonstrated greater interest in public health, reflecting the pressing societal needs in these areas and the strong commitment to addressing public health challenges where healthcare resources are limited (26, 35, 37). Moreover, the weaker effect of CPT for Academia (8.24%) in the mediation analysis, suggests that other factors, such as academic mentorship or research opportunities, may play a larger role. This is consistent with research suggesting that academic careers often evolve over time and are influenced by factors like mentorship and research interests rather than predefined career goals set early in one's education (34, 38).

The significant gender differences in the preference for business/entrepreneurship, where male students exhibited a stronger inclination, reflect traditional societal roles and expectations regarding financial independence and leadership, which persist across many regions despite evolving norms in the healthcare sector (21, 31, 39). These findings suggest that gender dynamics continue to influence career aspirations, even in fields where gender representation is becoming more balanced. Taken together, these results emphasise the need for culturally sensitive career guidance that considers sociocultural factors in shaping career goals and preferences.

### Career planning training

When career planning agencies and preferred media are considered, the logistic regression findings align well with students' stated preferences for dental schools as the most reliable CPT source, especially in high-income countries (23, 25, 28, 33). National and international associations such as the IADS were more favoured by students from low-income countries, underscoring the need for global networking and support systems for students with fewer local resources.

Interestingly, Gen Z students still preferred in-person training over digital platforms. This suggests that students highly value personal interaction with mentors and career advisors, even in an era of digital learning. This is supported by findings from Garcia et al., who investigated the effect of COVID-19 on the career perspective of dental and dental hygienist students—their findings concluded that according to student perception of the effect of the pandemic on their education, they reported a negative effect on the preclinical and clinical training pointing at the fatigue of virtual learning and the absence of hands-on practice (40).

The findings strongly support the idea that dental schools should integrate structured career counselling into their curricula to enhance students' self-efficacy and align their career goals with realistic professional expectations.

### Limitations

The findings confirm the applicability of SCCT in explaining dental students' career aspirations across diverse contexts. However, regional adaptations may be necessary to account for variations in professional and personal expectations, particularly in the socioeconomic context. Additionally, while this study provides significant insights into the role of Career Planning Training, it is limited by its cross-sectional design, which prevents us from drawing causal inferences. Future longitudinal studies could explore how changes in self-efficacy and professional expectations over time affect career choices. Furthermore, the overrepresentation of European participants and the underrepresentation of regions such as Asia-Pacific may limit the global generalizability of the findings. However, subgroup analyses and regression models were applied to account for regional differences, ensuring the results remain interpretable across diverse educational and professional contexts. Finally, while the SCCT components, i.e., self-efficacy, outcome expectations, and career goals, were examined, the influence of role models on career decisions was not assessed. Given the documented impact of mentorship on career trajectories, future research should incorporate this factor for a more comprehensive understanding of career aspirations.

### Implications

These findings have important implications for dental education and policy. National dental associations and schools should work collaboratively to create tailored career planning programs that address the unique needs of students in their pertaining regions. Moreover, organizations like FDI and IADS could focus on creating general CPT guidelines that can be adapted to local contexts, rather than providing CPT directly. Future research should continue exploring how CPT interventions can be tailored to students' specific career goals and regional job market conditions.

## Conclusions

This study offers new insights into the global career aspirations of dental students through the lens of the SCCT. The findings show that structured career planning programs can significantly enhance students' ability to make informed decisions about their future.

The differences in career aspirations based on income level and geographic region underline the need for more personalised career planning training, particularly in low-income areas where students may have fewer resources and less support. By increasing access to career planning training and focusing on building students' confidence and informed awareness of the available career opportunities, dental schools and associations can help ensure that students are better prepared for the diverse opportunities within the dental field.

## Data Availability

The data that support the findings of this study are available from the corresponding author upon reasonable request.

## Group members of IADS Delegates

Faculty of Dentistry, University of Medical Sciences and Technology, Khartoum, Sudan: Aya Abdelrahim. College of Dentistry, Kuwait University, Kuwait City, Kuwait: Yousif Alhasan. Faculty of Dentistry, Istanbul Medipol University, Istanbul, Türkiye: Selim Tunahan Akçay. Faculty of Dentistry, Hacettepe University, Ankara, Türkiye: Osman Çaksen. Faculty of Dentistry, Aldent University, Tirana, Albania: Ersid Domnori. Faculty of Dentistry, Universidad de San Carlos de Guatemala, Guatemala City, Guatemala: Andrea Martinez, Gabriela Molina, Juan Fernando Morales. Faculty of Dentistry, Alamein International University, New Alamein City, Egypt: Aya Eida. Faculty of Dentistry, Alexandria University, Alexandria, Egypt: Gamal ElGhonemy. Faculty of Dentistry, Al Neelain University, Khartoum, Sudan: Zeinab Hassan. Faculty of Dentistry, University of Khartoum, Khartoum, Sudan: Rawan Mahmoud. Faculty of Dentistry, University of Medicine, Tirana, Albania: Eni Shaqiri. Faculty of Dentistry, Istanbul Medipol University, Istanbul, Türkiye: Zeynep Önen.

## Group members of EDSA Delegates

Faculty of Dental Surgery, Nantes Université, Nantes, France: Inès Bouillaud. School of Dental Medicine, University of Zagreb, Zagreb, Croatia: Klara Križan, Matija Borovac. School of Dental Medicine, University of Bern, Bern, Switzerland: Yanik Bossi. Faculty of Dentistry, University of Porto, Porto, Portugal: Diana Correia. Oral & Maxillofacial Surgery Department, University Clinical Center of Kosovo: Behar Dalipi. Faculty of Dental Medicine, Medical University of Plovdiv, Plovdiv, Bulgaria: Ivan Demyanov. Faculty of Dentistry, University of Coimbra, Coimbra, Portugal: Bianca Gomes. Faculty of Dentistry, Riga Stradiņš University, Riga, Latvia: Kristīne Romanovska, Luīze Lote Liepiņa, Paula Paegle. Faculty of Medicine, University of Ljubljana, Ljubljana, Slovenia: Tjaša Miklavčič. Faculty of Medicine, Charles University, Pilsen, Czech Republic: Jana Panochová. University Center for Dental Medicine Basel, University of Basel, Basel, Switzerland: Mark RutscheidtCenter of Dental Medicine, University of Zurich, Zurich, Switzerland: Bogomil Sabev. Faculty of Dentistry, University of Lisbon, Lisbon, Portugal: Mafalda Silva. Faculty of Dental Medicine, University of Rijeka, Rijeka, Croatia: Elizabeta Vrkljan.
