# Supplementary material for: Career aspirations of dental students: insights from a multinational study using social cognitive career theory (SCCT)
Source: Front Oral Health. 2025 Apr 11;6:1577870. doi: 10.3389/froh.2025.1577870 (PMC12021817; doi:10.3389/froh.2025.1577870)

**Table S1.** Questionnaire of Dental Students’ Career Aspirations Used in the FDI-IADS-EDSA Survey of Career Planning

| **#** | **Personal Information** | | | | | |
| --- | --- | --- | --- | --- | --- | --- |
| 1 | Gender | - Female - Male - Other (please specify) - Prefer not to say | | | | |
| 2 | Age | - Dropdown menu of numbers (17-99) | | | | |
| 3 | Academic Year | - 1^st^ Year - 2^nd^ Year - 3^rd^ Year - 4^th^ Year - 5^th^ Year - 6^th^ Year - Intern (Vocational Training) - Graduated, please specify the graduation date (mm/yyyy) | | | | |
| 4 | Nationality | - Dropdown menu | | | | |
| 5 | Country of Study | - Dropdown menu | | | | |
| 6 | University Type | - Public (state-funded) - Private (self-funded) | | | | |
| 7 | Student Loan | - Yes - No | | | | |
|  | **How confident are you at this stage of your training that you could…** | | | | | |
| 8 | choose a career path that will fulfil your **expectations and goals**. | Strongly disagree | Disagree a little | Neither agree nor disagree | Agree a little | Strongly agree |
| 9 | choose a career path that will fit well with your **personality** (e.g., being an extrovert/introvert). | Strongly disagree | Disagree a little | Neither agree nor disagree | Agree a little | Strongly agree |
| 10 | choose a career path that will enable you to live the type of **lifestyle** you desire. | Strongly disagree | Disagree a little | Neither agree nor disagree | Agree a little | Strongly agree |
| 11 | choose a career path that will fit your **interests and abilities**. | Strongly disagree | Disagree a little | Neither agree nor disagree | Agree a little | Strongly agree |
| 12 | decide what you are and are not ready to **sacrifice** in order to choose a career path. | Strongly disagree | Disagree a little | Neither agree nor disagree | Agree a little | Strongly agree |
| 13 | decide what you **value** most in a medical career (e.g., relationships with patients, prestige, or technical skills, etc.). | Strongly disagree | Disagree a little | Neither agree nor disagree | Agree a little | Strongly agree |
| 14 | locate valid and accurate **information** to help you choose between equally desirable specialities. | Strongly disagree | Disagree a little | Neither agree nor disagree | Agree a little | Strongly agree |
|  | **When thinking about the type of career path you are interested in (e.g., clinical practice, academia, public health), how much do you expect at this stage of your training that your choice of career path will…** | | | | | |
| 15 | be **intellectually** stimulating. | Strongly disagree | Disagree a little | Neither agree nor disagree | Agree a little | Strongly agree |
| 16 | provide you with work **satisfaction**. | Strongly disagree | Disagree a little | Neither agree nor disagree | Agree a little | Strongly agree |
| 17 | allow you to interact with your **colleagues**. | Strongly disagree | Disagree a little | Neither agree nor disagree | Agree a little | Strongly agree |
| 18 | let you practice clinical **skills** that best suit your perceived abilities. | Strongly disagree | Disagree a little | Neither agree nor disagree | Agree a little | Strongly agree |
| 19 | provide you with a good **income**. | Strongly disagree | Disagree a little | Neither agree nor disagree | Agree a little | Strongly agree |
| 20 | allow you to perform a **broad** **spectrum** of work. | Strongly disagree | Disagree a little | Neither agree nor disagree | Agree a little | Strongly agree |
| 21 | be compatible with your **interests**. | Strongly disagree | Disagree a little | Neither agree nor disagree | Agree a little | Strongly agree |
| 22 | allow you to achieve your desired professional **success**. | Strongly disagree | Disagree a little | Neither agree nor disagree | Agree a little | Strongly agree |
|  | **When thinking about the type of career path you are interested in (e.g., clinical practice, academia, public health), how much do you expect at this stage of your training that your choice of career path will…** | | | | | |
| 23 | allow you to work the **number of** **hours** that you desire. | Strongly disagree | Disagree a little | Neither agree nor disagree | Agree a little | Strongly agree |
| 24 | allow you to pursue **leisure** time activities/interests that you like. | Strongly disagree | Disagree a little | Neither agree nor disagree | Agree a little | Strongly agree |
| 25 | allow you to have your desired work/recreational **balance**. | Strongly disagree | Disagree a little | Neither agree nor disagree | Agree a little | Strongly agree |
| 26 | allow you to have your desired **lifestyle**. | Strongly disagree | Disagree a little | Neither agree nor disagree | Agree a little | Strongly agree |
| ^[2]^ | **When you think about the type of career path that you might choose (e.g., clinical practice, academia, public health), please indicate if, at this stage of your training, you agree or disagree with the following statements:** | | | | | |
| 27 | I have a **clear set of goals** for my future with regard to choosing a career path. | Strongly disagree | Disagree a little | Neither agree nor disagree | Agree a little | Strongly agree |
| 28 | I have discussed my goals in relation to my career path choice with my **family/partner**. | Strongly disagree | Disagree a little | Neither agree nor disagree | Agree a little | Strongly agree |
| 29 | I am taking the **steps needed** to achieve my goal of choosing a career path. | Strongly disagree | Disagree a little | Neither agree nor disagree | Agree a little | Strongly agree |
| 30 | I have examined my **interests, values, and abilities** in detail to come up with my goal of choosing a career path. | Strongly disagree | Disagree a little | Neither agree nor disagree | Agree a little | Strongly agree |
| 31 | I have a set **time frame** in which to make a decision about my choice of career path. | Strongly disagree | Disagree a little | Neither agree nor disagree | Agree a little | Strongly agree |
| 32 | I am getting lots of **support** to achieve my goal of choosing a career path. | Strongly disagree | Disagree a little | Neither agree nor disagree | Agree a little | Strongly agree |
|  | **Career Planning Training** | | | | | |
| 34 | Have you been given enough information during your undergraduate study to make an informed decision about your professional career? ^[8]^ | - Yes - No - Not Sure | | | | |
| 35 | Which career path is your **first preference** upon graduation? | - Clinical Dentistry Career Pathway (General) - Clinical Dentistry Career Pathway (Specialist) - Academic Career Pathway - Public Health Career Pathway (e.g., NGO, IGO) - Business/Entrepreneurship Career Pathway - I am not sure yet | | | | |
| 36 | *Which career path is your* ***second preference*** *upon graduation?* | - Clinical Dentistry Career Pathway (General) - Clinical Dentistry Career Pathway (Specialist) - Academic Career Pathway - Public Health Career Pathway (e.g., NGO, IGO) - Business/Entrepreneurship Career Pathway - I am not sure yet | | | | |
| 37 | In your opinion, which organisation(s) should be responsible for providing career guidance and information to dental students as they plan their professional futures? | - Dental Schools (University) - World Dental Federation (FDI) - Regional Dental Associations (e.g., FDI-ERO, CED) - National Dental Associations - International Dental Students Association (IADS) - Regional Dental Students Associations (e.g., EDSA, APDSA, AfroDSA) - National Dental Students Associations | | | | |
| 38 | What types of information source(s) do you prefer to use for planning your dental career? | - Virtual Sources: Webinars - Virtual Sources: Podcasts - Virtual Sources: Blogs - Virtual Sources: E-books - In-person Sources: Continuous education workshops - In-person Sources: Elective courses in dental schools (undergraduate training) | | | | |
| 39 | At what point in their undergraduate education do you think dental students should be introduced to information about various career paths in dentistry?^[9]^ | - Upon entering dental school (e.g., first-year students) - At the beginning of clinical years (e.g., third-year students) - Towards the end of dental school (e.g., senior students) | | | | |
| 40 | Do you think continuous education programs (CE) should have components about career management? | - Yes - No - Not Sure | | | | |

**Table S2.** Nationality and Study Country of Dental Students Participating in the FDI–IADS–EDSA Survey on Career Planning, May – July 2023 (*n* = 1964)

| **Nationality** | | | **Study** | | |  |
| --- | --- | --- | --- | --- | --- | --- |
| **Country** | **Region** | **Frequency (%)** | **Country** | **Region** | **Frequency (%)** | |
| Portugal | EU | 187 (9.52%) | Portugal | EU | 277 (14.10%) | |
| Albania | EU | 165 (8.40%) | Switzerland | EU | 166 (8.45%) | |
| France | EU | 155 (7.89%) | Bulgaria | EU | 156 (7.94%) | |
| Switzerland | EU | 145 (7.38%) | Albania | EU | 140 (7.13%) | |
| Bulgaria | EU | 136 (6.92%) | Guatemala | SA | 123 (6.26%) | |
| Guatemala | SA | 122 (6.21%) | Egypt | AF | 102 (5.19%) | |
| Egypt | AF | 97 (4.94%) | France | EU | 84 (4.28%) | |
| Czech Republic | EU | 67 (3.41%) | Czech Republic | EU | 81 (4.12%) | |
| Germany | EU | 64 (3.26%) | Kosovo | EU | 77 (3.92%) | |
| Sudan | AF | 59 (3%) | Croatia | EU | 59 (3%) | |
| Croatia | EU | 56 (2.85%) | Sudan | AF | 58 (2.95%) | |
| Kosovo | EU | 55 (2.80%) | Germany | EU | 56 (2.85%) | |
| Turkey | EU | 55 (2.80%) | Latvia | EU | 56 (2.85%) | |
| Latvia | EU | 54 (2.75%) | Turkey | EU | 56 (2.85%) | |
| Slovenia | EU | 47 (2.39%) | Slovenia | EU | 48 (2.44%) | |
| Jordan | AP | 40 (2.04%) | Kuwait | AP | 39 (1.99%) | |
| Morocco | AF | 40 (2.04%) | Morocco | AF | 39 (1.99%) | |
| Kuwait | AP | 38 (1.93%) | Tunisia | AF | 37 (1.88%) | |
| Tunisia | AF | 36 (1.83%) | Indonesia | AP | 35 (1.78%) | |
| Indonesia | AP | 35 (1.78%) | Jordan | AP | 35 (1.78%) | |
| Palestine | AP | 29 (1.48%) | Palestine | AP | 29 (1.48%) | |
| Lebanon | AP | 25 (1.27%) | Lithuania | EU | 27 (1.37%) | |
| Lithuania | EU | 19 (0.97%) | Lebanon | AP | 25 (1.27%) | |
| Mexico | NA | 18 (0.92%) | Mexico | NA | 19 (0.97%) | |
| Estonia | EU | 17 (0.87%) | Estonia | EU | 18 (0.92%) | |
| Kenya | AF | 15 (0.76%) | Rwanda | AF | 16 (0.81%) | |
| Nigeria | AF | 15 (0.76%) | Kenya | AF | 15 (0.76%) | |
| Rwanda | AF | 15 (0.76%) | Nigeria | AF | 15 (0.76%) | |
| Slovakia | EU | 14 (0.71%) | Poland | EU | 13 (0.66%) | |
| North Macedonia | EU | 12 (0.61%) | North Macedonia | EU | 11 (0.56%) | |
| United Kingdom | EU | 12 (0.61%) | Israel | EU | 8 (0.41%) | |
| Italy | EU | 9 (0.46%) | India | AP | 5 (0.25%) | |
| Syria | AP | 9 (0.46%) | Brazil | SA | 4 (0.20%) | |
| Brazil | SA | 7 (0.36%) | Slovakia | EU | 4 (0.20%) | |
| India | AP | 6 (0.31%) | United Kingdom | EU | 4 (0.20%) | |
| Iran | AP | 6 (0.31%) | Italy | EU | 3 (0.15%) | |
| Israel | EU | 6 (0.31%) | Pakistan | AP | 3 (0.15%) | |
| Bosnia and Herzegovina | EU | 5 (0.25%) | Romania | EU | 3 (0.15%) | |
| Poland | EU | 5 (0.25%) | Serbia | EU | 3 (0.15%) | |
| Pakistan | AP | 4 (0.20%) | Canada | NA | 2 (0.10%) | |
| Russia | EU | 4 (0.20%) | Cyprus | EU | 2 (0.10%) | |
| Serbia | EU | 4 (0.20%) | Bosnia and Herzegovina | EU | 1 (0.05%) | |
| Greece | EU | 3 (0.15%) | Chile | SA | 1 (0.05%) | |
| Ireland | EU | 3 (0.15%) | Iran | AP | 1 (0.05%) | |
| Romania | EU | 3 (0.15%) | Iraq | AP | 1 (0.05%) | |
| Spain | EU | 3 (0.15%) | Ireland | EU | 1 (0.05%) | |
| Algeria | AF | 2 (0.10%) | Netherlands | EU | 1 (0.05%) | |
| Angola | AF | 2 (0.10%) | Russia | EU | 1 (0.05%) | |
| Austria | EU | 2 (0.10%) | Spain | EU | 1 (0.05%) | |
| Belgium | EU | 2 (0.10%) | Sri Lanka | AP | 1 (0.05%) | |
| Canada | NA | 2 (0.10%) | Taiwan | AP | 1 (0.05%) | |
| Chile | SA | 2 (0.10%) | Tanzania | AF | 1 (0.05%) | |
| Finland | EU | 2 (0.10%) |  | | |  |
| Iraq | AP | 2 (0.10%) |  |  |  |  |
| Liechtenstein | EU | 2 (0.10%) |  |  |  |  |
| Mauritania | AF | 2 (0.10%) |  |  |  |  |
| Netherlands | EU | 2 (0.10%) |  |  |  |  |
| Norway | EU | 2 (0.10%) |  |  |  |  |
| Taiwan | AP | 2 (0.10%) |  |  |  |  |
| United States | NA | 2 (0.10%) |  |  |  |  |
| Armenia | AP | 1 (0.05%) |  |  |  |  |
| Azerbaijan | AP | 1 (0.05%) |  |  |  |  |
| Belarus | EU | 1 (0.05%) |  |  |  |  |
| Burundi | AF | 1 (0.05%) |  |  |  |  |
| Cuba | SA | 1 (0.05%) |  |  |  |  |
| Cyprus | EU | 1 (0.05%) |  |  |  |  |
| Denmark | EU | 1 (0.05%) |  |  |  |  |
| Gabon | AF | 1 (0.05%) |  |  |  |  |
| Kazakhstan | AP | 1 (0.05%) |  |  |  |  |
| Moldova | EU | 1 (0.05%) |  |  |  |  |
| Montenegro | EU | 1 (0.05%) |  |  |  |  |
| South Sudan | AF | 1 (0.05%) |  |  |  |  |
| Sri Lanka | AP | 1 (0.05%) |  |  |  |  |
| Tanzania | AF | 1 (0.05%) |  |  |  |  |
| Ukraine | EU | 1 (0.05%) |  |  |  |  |

Africa = AF, Asia-Pacific = AP, Europe = EU, North America = NA, South America = SA

**Table S3.** Overall Scores of Self-efficacy, Professional Expectations, Personal Expectations and Career Goals among Dental Students Participating in FDI–IADS–EDSA Survey of Career Planning, Stratified by Career Preferences, and Career Planning Training Agencies and Preferred Media, May – July 2023, (*n* = 1964)

| **Variable** | **Outcome** | **Self-efficacy** | ***p*.** | **Professional Expectations** | ***p*.** | **Personal Expectations** | ***p*.** | **Career Goals** | ***p*.** |
| --- | --- | --- | --- | --- | --- | --- | --- | --- | --- |
| **First Preference** | Clinical Dentistry (General) | 26.99 ± 4.28 | **<0.001** | 32.40 ± 4.63 | **<0.001** | 14.99 ± 3.31 | **0.046** | 22.02 ± 3.83 | **<0.001** |
|  | Clinical Dentistry (Specialty) | 27.20 ± 4.47 |  | 32.81 ± 4.96 |  | 14.81 ± 3.55 |  | 22.29 ± 3.76 |  |
|  | Business / Entrepreneurship | 25.34 ± 5.24 |  | 30.35 ± 6.30 |  | 13.63 ± 4.12 |  | 21.10 ± 4.27 |  |
|  | Academia | 26.69 ± 5.31 |  | 31.84 ± 6.14 |  | 14.46 ± 3.81 |  | 21.96 ± 3.80 |  |
|  | Public Health | 25.18 ± 5.88 |  | 30.41 ± 7.16 |  | 14.36 ± 4.35 |  | 20.80 ± 4.59 |  |
|  | Undecided | 24.92 ± 5.26 |  | 30.95 ± 5.81 |  | 14.44 ± 3.68 |  | 20.51 ± 4.18 |  |
| **Second Preference** | Clinical Dentistry (General) | 26.83 ± 4.45 | **0.037** | 32.50 ± 5.03 | **0.046** | 14.73 ± 3.67 | 0.330 | 22.26 ± 3.69 | 0.053 |
|  | Clinical Dentistry (Specialty) | 26.72 ± 4.53 |  | 32.13 ± 5.10 |  | 14.79 ± 3.51 |  | 22.12 ± 3.72 |  |
|  | Business / Entrepreneurship | 27.61 ± 4.33 |  | 33.24 ± 4.53 |  | 15.28 ± 3.44 |  | 22.72 ± 3.48 |  |
|  | Academia | 27.49 ± 4.24 |  | 33.04 ± 4.83 |  | 14.75 ± 3.41 |  | 21.86 ± 3.93 |  |
|  | Public Health | 27.38 ± 4.72 |  | 32.52 ± 5.27 |  | 14.76 ± 3.50 |  | 21.25 ± 4.17 |  |
|  | Undecided | 26.49 ± 5.01 |  | 31.85 ± 5.64 |  | 14.51 ± 3.64 |  | 21.84 ± 4.30 |  |
| **Suggested Agencies** | Dental Schools | 26.75 ± 4.61 | 0.464 | 32.41 ± 5.06 | 0.051 | 14.81 ± 3.52 | **0.050** | 21.94 ± 3.87 | 0.859 |
|  | FDI | 26.85 ± 4.91 | 0.346 | 32.45 ± 5.77 | **0.030** | 14.60 ± 3.84 | 0.583 | 22.06 ± 3.94 | 0.187 |
|  | Reg. Dent. Assoc. | 26.84 ± 4.64 | 0.877 | 32.46 ± 5.19 | 0.578 | 14.56 ± 3.74 | 0.222 | 21.81 ± 3.86 | 0.275 |
|  | Nat. Dent. Assoc. | 26.61 ± 4.77 | 0.238 | 32.53 ± 5.13 | 0.140 | 14.65 ± 3.64 | 0.311 | 21.67 ± 3.87 | **0.004** |
|  | IADS | 26.73 ± 4.67 | 0.627 | 32.57 ± 5.28 | 0.068 | 14.44 ± 3.74 | **0.012** | 21.80 ± 4.00 | 0.438 |
|  | Reg. Dent. Stud. Assoc. | 26.77 ± 4.82 | 0.793 | 32.70 ± 5.38 | **0.004** | 14.74 ± 3.58 | 0.889 | 21.70 ± 3.95 | 0.124 |
|  | Nat. Dent. Stud. Assoc. | 26.70 ± 4.77 | 0.736 | 32.69 ± 5.12 | **0.004** | 14.75 ± 3.64 | 0.948 | 21.88 ± 3.90 | 0.525 |
| **Information Sources** | Webinars | 26.61 ± 4.60 | 0.121 | 32.36 ± 5.04 | 0.981 | 14.72 ± 3.60 | 0.715 | 21.90 ± 3.88 | 0.606 |
|  | Podcasts | 26.42 ± 4.73 | **0.047** | 32.32 ± 5.00 | 0.972 | 14.78 ± 3.60 | 0.516 | 21.88 ± 3.89 | 0.703 |
|  | Blogs | 26.92 ± 4.59 | 0.382 | 32.60 ± 4.97 | 0.237 | 14.83 ± 3.56 | 0.506 | 21.88 ± 3.89 | 0.662 |
|  | E-books | 26.96 ± 4.73 | 0.166 | 32.67 ± 4.80 | 0.307 | 14.97 ± 3.37 | 0.227 | 22.18 ± 3.82 | 0.182 |
|  | CE Workshops | 26.78 ± 4.58 | 0.931 | 32.49 ± 4.99 | 0.181 | 14.71 ± 3.51 | 0.378 | 21.97 ± 3.82 | 0.859 |
|  | UG Courses | 26.77 ± 4.72 | 0.860 | 32.47 ± 5.26 | **0.043** | 14.77 ± 3.60 | 0.641 | 21.88 ± 3.91 | 0.489 |
| **Undergraduate Education** | Freshman Years | 27.03 ± 4.96 | 0.058 | 32.46 ± 5.39 | 0.161 | 14.99 ± 3.60 | **0.044** | 22.23 ± 3.86 | **0.045** |
|  | Middle Years | 26.65 ± 4.56 |  | 32.30 ± 5.10 |  | 14.68 ± 3.54 |  | 21.78 ± 3.90 |  |
|  | Senior Years | 26.50 ± 4.33 |  | 31.99 ± 5.01 |  | 14.46 ± 3.53 |  | 21.72 ± 4.02 |  |
| **Continuous Education** | Yes | 26.54 ± 4.30 | 0.762 | 32.15 ± 5.18 | 0.590 | 14.19 ± 3.88 | 0.411 | 22.44 ± 3.84 | **0.028** |
|  | Unsure | 26.48 ± 4.13 |  | 31.61 ± 4.92 |  | 15.13 ± 2.99 |  | 20.61 ± 4.55 |  |
|  | No | 24.67 ± 5.89 |  | 27.67 ± 10.69 |  | 12.50 ± 4.14 |  | 18.83 ± 5.23 |  |

Mann-Whitney (*U*) and Kruskal-Wallis (*H*) tests were used with a significance level (*p*.) < 0.05.

**Figure S1.** Flowchart of Responses Received and Included in the FDI-IADS-EDSA Survey on Career Planning, May – July 2023


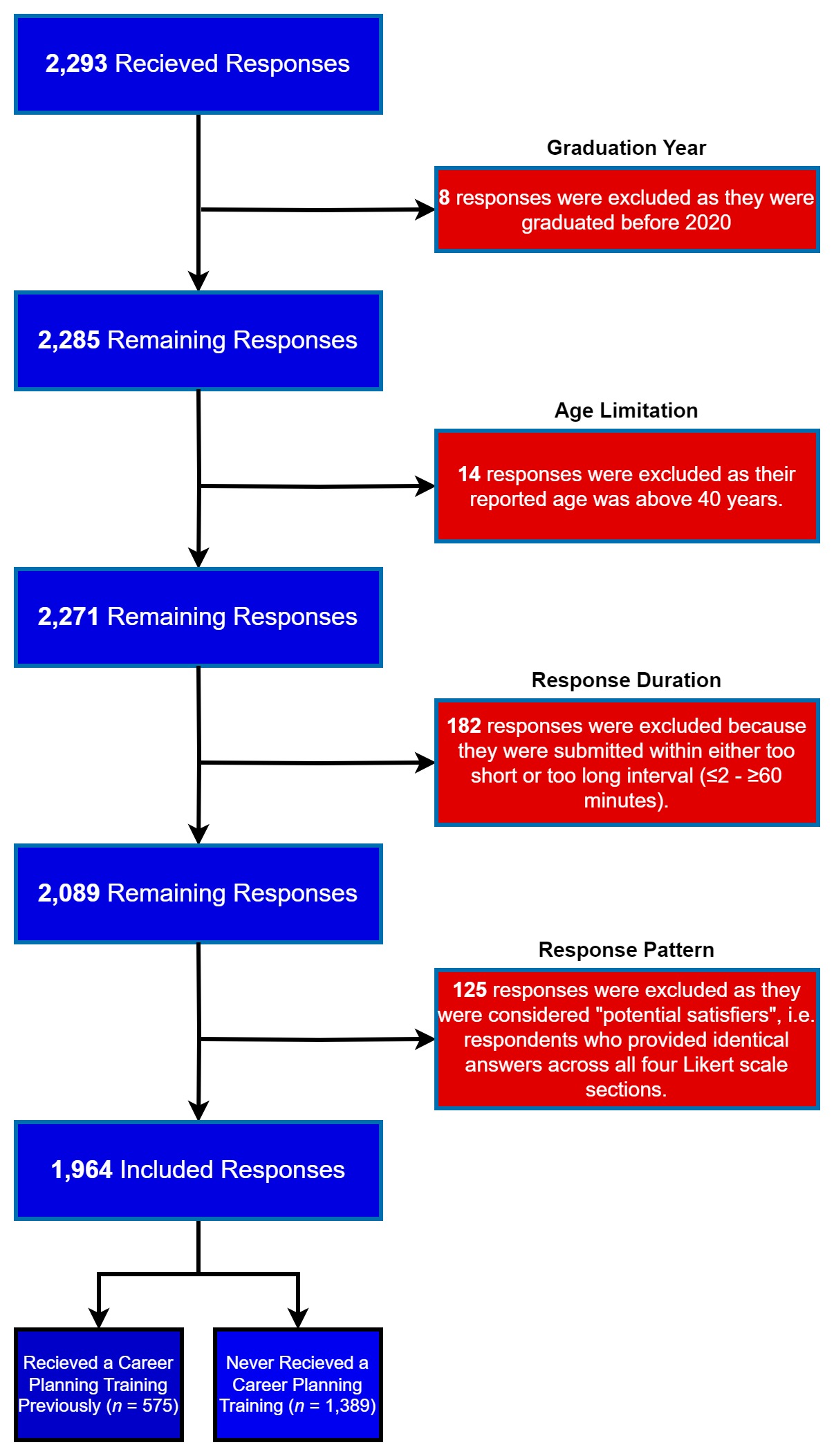

Supplement: Supplementary file 1 [file Table1.docx]
