# Supplementary figures and images for: Career aspirations of dental students: insights from a multinational study using social cognitive career theory (SCCT)
Source: Front Oral Health. 2025 Apr 11;6:1577870. doi: 10.3389/froh.2025.1577870 (PMC12021817; doi:10.3389/froh.2025.1577870)

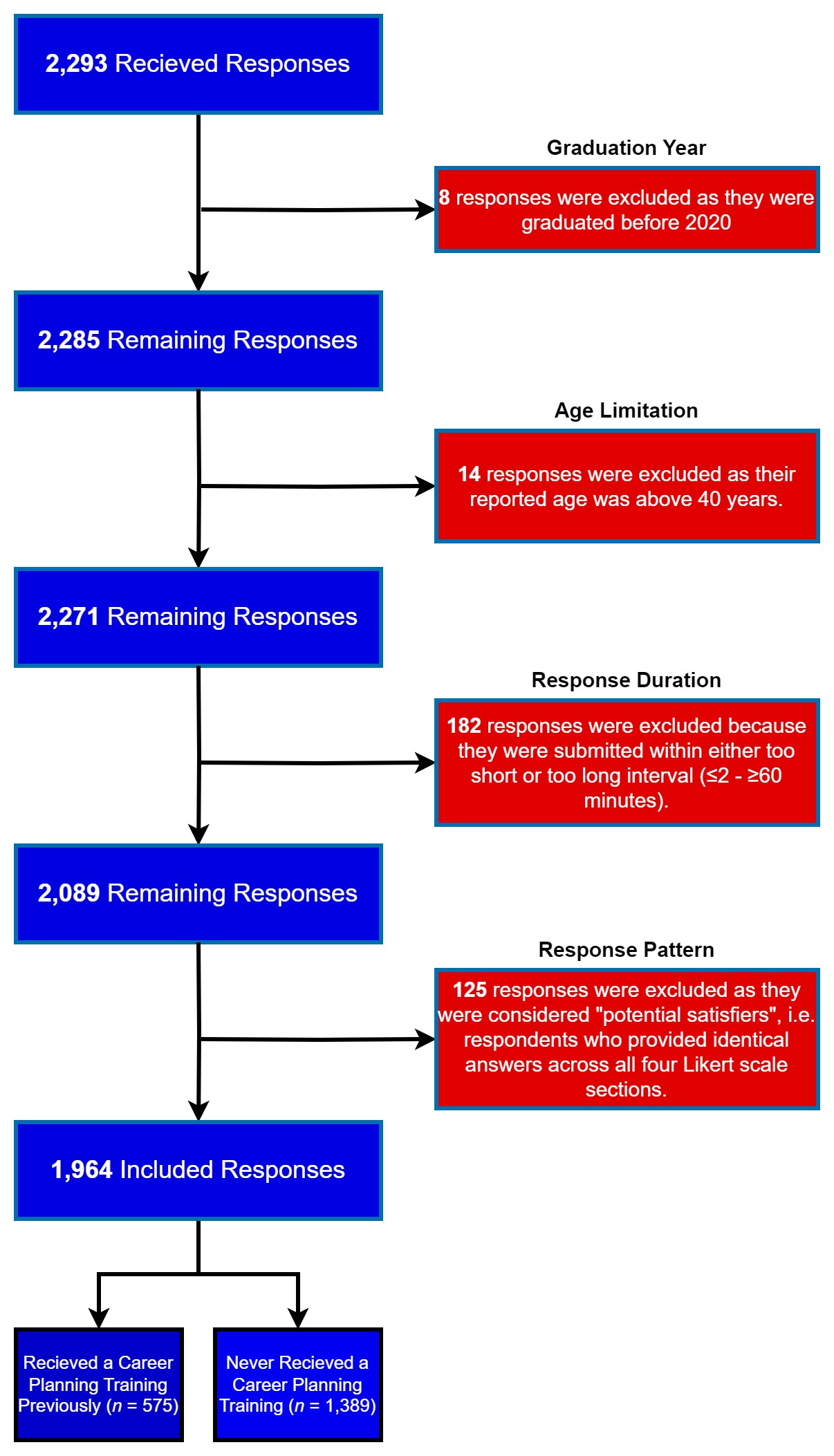

Supplement: Supplementary file 2 [file Image1.jpeg]
